# Supplementary material for: Burden of acute lymphoblastic leukemia in children and adolescents in low- and middle-income countries from 1990 to 2023 and projections to 2050: A systematic analysis from the global burden of disease study 2023
Source: PLoS One. 2026 Jun 2;21(6):e0350223. doi: 10.1371/journal.pone.0350223 (PMC13229300; doi:10.1371/journal.pone.0350223)
Supplement: S1 Table — (DOCX) [file pone.0350223.s001.docx]

| S1 Table. Incidence, deaths, and DALYs number of acute lymphoblastic leukemia in 2023 | | | | |
| --- | --- | --- | --- | --- |
| Locations | Sex | Incidence.number..95.UI | Death.number..95.UI | DALYs.number..95.UI |
| World Bank Upper Middle Income | Male | 38373.6 (28425.9 to 53787) | 18355.3 (15587.4 to 22718.4) | 468920.1 (401632.8 to 577988.4) |
| World Bank Upper Middle Income | Female | 15134.6 (9726.2 to 22170.5) | 6750.5 (4763.7 to 8552.7) | 158639.2 (113482 to 201451) |
| World Bank Upper Middle Income | Both | 53508.2 (39858.3 to 73422.6) | 25105.8 (21339.6 to 29728.1) | 627559.3 (541609 to 742179.7) |
| World Bank Lower Middle Income | Male | 9672.7 (6392.2 to 13692.3) | 8848.4 (5772.2 to 12543) | 232851.2 (152323.6 to 328014.2) |
| World Bank Lower Middle Income | Female | 5052.1 (3262.2 to 7742.5) | 4512.8 (2970.8 to 6858.3) | 114239.6 (74851.2 to 174536.4) |
| World Bank Lower Middle Income | Both | 14724.8 (10617.5 to 19331.5) | 13361.1 (9811.5 to 17337) | 347090.7 (254272.7 to 447925) |
| World Bank Low Income | Male | 856.6 (511.6 to 1225.9) | 816.4 (481.9 to 1173.3) | 21765.6 (12867.8 to 31208.5) |
| World Bank Low Income | Female | 605.6 (388.2 to 947.9) | 573.3 (374.3 to 906.4) | 14690.7 (9560.3 to 22806) |
| Algeria | Females | 6.1 (2.8 to 12.6) | 65.3 (39.2 to 98.7) | 8097 (4678.2 to 12204.3) |
| Algeria | Both sexes | 14.7 (7.8 to 25.8) | 160.5 (102.7 to 238.1) | 13334.6 (8475 to 20602.8) |
| Angola | Males | 1510 (875.1 to 2430.6) | 258.4 (169.3 to 375.4) | 835.4 (681 to 999.2) |
| Angola | Females | 1026.3 (519.5 to 1689.3) | 145.1 (85.7 to 233.6) | 773.9 (610.7 to 981) |
| Angola | Both sexes | 2536.3 (1524.9 to 3737.2) | 403.5 (282.9 to 564.9) | 1609.3 (1380.6 to 1834.5) |
| Argentina | Males | 4.7 (3.1 to 6.6) | 90.7 (73.3 to 106.4) | 10.8 (6.4 to 17.8) |
| Argentina | Females | 3.9 (2.5 to 6.3) | 58.6 (48.7 to 70.1) | 24.7 (15.6 to 38.7) |
| Argentina | Both sexes | 8.5 (5.9 to 12.8) | 149.3 (129.5 to 168.4) | 35.5 (24.6 to 50.7) |
| Armenia | Males | 620.9 (328.9 to 926.2) | 3.7 (2.8 to 4.8) | 545.6 (133.2 to 1643.5) |
| Armenia | Females | 496.5 (277.7 to 798.8) | 2.4 (1.8 to 3) | 1376.1 (719.7 to 2519.9) |
| Armenia | Both sexes | 1117.4 (725.5 to 1549.8) | 6.1 (5 to 7.4) | 1921.6 (995.3 to 3468.3) |
| Azerbaijan | Males | 28.2 (20.8 to 40) | 48.3 (25.8 to 73.7) | 12.9 (9.3 to 16.9) |
| Azerbaijan | Females | 15.8 (10.7 to 22.5) | 25.5 (15.1 to 37.9) | 7.5 (5.6 to 9.9) |
| Azerbaijan | Both sexes | 44 (34.5 to 60.1) | 73.8 (44.9 to 103.6) | 20.4 (16.3 to 25.2) |
| Bangladesh | Males | 0 (0 to 0) | 401.9 (230.5 to 626) | 11800.9 (6107.1 to 17755) |
| Bangladesh | Females | 0.1 (0 to 0.1) | 241.6 (140.1 to 394.7) | 7409.5 (4704 to 10954.4) |
| Bangladesh | Both sexes | 0.1 (0 to 0.1) | 643.5 (420.4 to 945.6) | 19210.3 (12004 to 26442.3) |
| Belarus | Males | 0 (0 to 0.1) | 3.8 (2.7 to 5) | 2257.4 (1818.2 to 2792.2) |
| Belarus | Females | 0.1 (0 to 0.1) | 3.3 (2.4 to 4.4) | 1706.6 (1302.9 to 2232.5) |
| Belarus | Both sexes | 0.1 (0.1 to 0.2) | 7.1 (5.8 to 8.7) | 3964 (3303.5 to 4843.7) |
| Belize | Males | 84.1 (42 to 166.1) | 0.9 (0.7 to 1.1) | 18242.9 (10260 to 30904.7) |
| Belize | Females | 63.3 (35 to 112) | 0.7 (0.5 to 0.8) | 13237.5 (7467.2 to 21066.9) |
| Belize | Both sexes | 147.4 (86.1 to 252.2) | 1.5 (1.3 to 1.8) | 31480.4 (20594.1 to 46589.5) |
| Benin | Males | 24 (16.7 to 35.6) | 136.2 (83.4 to 212.7) | 2478.7 (1277.1 to 4026) |
| Benin | Females | 11.9 (7.6 to 21.1) | 90.4 (56.4 to 140.7) | 1933.3 (1225.6 to 2842.3) |
| Benin | Both sexes | 35.8 (25.6 to 55.5) | 226.6 (156.7 to 327.4) | 4412.1 (2857.4 to 6492) |
| Bhutan | Males | 85.3 (46.9 to 133.8) | 1.7 (1 to 2.8) | 5598.5 (2728.9 to 9056.2) |
| Bhutan | Females | 65.1 (38.5 to 103.2) | 1.1 (0.6 to 1.8) | 4229.2 (2604.4 to 6575.9) |
| Bhutan | Both sexes | 150.3 (101.7 to 222.8) | 2.8 (1.8 to 4.2) | 9827.7 (5724.9 to 14834.1) |
| Bolivia (Plurinational State of) | Males | 204.2 (90.7 to 411.2) | 65.7 (39.4 to 100.8) | 68.6 (55 to 87.3) |
| Bolivia (Plurinational State of) | Females | 140.3 (63.4 to 282.2) | 69.9 (44.3 to 109) | 54 (43.2 to 65.7) |
| Bolivia (Plurinational State of) | Both sexes | 344.5 (181.1 to 631.3) | 135.6 (90.8 to 198.9) | 122.6 (103.1 to 143.2) |
| Bosnia and Herzegovina | Males | 91.4 (47.1 to 147.4) | 2 (1.1 to 3.3) | 691.6 (424.7 to 1047.4) |
| Bosnia and Herzegovina | Females | 47.4 (26.9 to 79.3) | 1.1 (0.7 to 1.7) | 587.6 (369.3 to 875.4) |
| Bosnia and Herzegovina | Both sexes | 138.8 (81.5 to 214.8) | 3.1 (1.9 to 4.7) | 1279.2 (879.4 to 1780.2) |
| Botswana | Males | 10.1 (2.4 to 29.7) | 3.9 (2 to 6.4) | 4326.3 (1788.4 to 7240) |
| Botswana | Females | 27.6 (14.3 to 50.9) | 7.1 (4.4 to 10.8) | 4140 (2042.8 to 6594.5) |
| Botswana | Both sexes | 37.7 (20 to 70.9) | 11 (7.1 to 15.9) | 8466.3 (4495 to 12450.9) |
| Brazil | Males | 336.5 (191.6 to 558.7) | 338.8 (301.1 to 384.3) | 7870.8 (4403.3 to 12810) |
| Brazil | Females | 250.4 (140.1 to 404.8) | 268.2 (233.3 to 305.3) | 5420.2 (3256.6 to 8239.4) |
| Brazil | Both sexes | 586.9 (391.7 to 876.1) | 607 (548.4 to 674.9) | 13291 (8526.1 to 19556) |
| Burkina Faso | Males | 0 (0 to 0.1) | 69.5 (43.3 to 112.3) | 2407 (1323.9 to 4087.6) |
| Burkina Faso | Females | 0.3 (0.1 to 0.5) | 60.7 (40.6 to 91.5) | 1668.4 (981.4 to 2551.6) |
| Burkina Faso | Both sexes | 0.3 (0.2 to 0.5) | 130.3 (89.3 to 191) | 4075.4 (2711.9 to 6490.5) |
| Burundi | Males | 103.7 (43 to 186.8) | 85.4 (39.3 to 149.7) | 317.9 (225.5 to 421.1) |
| Burundi | Females | 107.3 (53.5 to 187.4) | 64.6 (37.7 to 97.4) | 270.2 (197.7 to 360) |
| Burundi | Both sexes | 210.9 (116.2 to 362.2) | 150 (83.1 to 224.2) | 588.1 (473.9 to 728) |
| Cabo Verde | Males | 52.6 (27.5 to 108.7) | 1.5 (0.8 to 2.9) | 6880.5 (3331.3 to 11426.3) |
| Cabo Verde | Females | 35 (20.1 to 55.9) | 0.7 (0.4 to 1.2) | 4015.4 (2260.2 to 5915.1) |
| Cabo Verde | Both sexes | 87.7 (56.6 to 155.3) | 2.2 (1.4 to 3.6) | 10895.9 (6191.4 to 16332.7) |
| Cambodia | Males | 0.3 (0.1 to 0.7) | 57.4 (32.5 to 91.4) | 7256.9 (5862.4 to 8494) |
| Cambodia | Females | 1.2 (0.6 to 2) | 41.3 (24.3 to 66.1) | 4729.8 (3921.6 to 5624) |
| Cambodia | Both sexes | 1.4 (0.8 to 2.5) | 98.6 (65.8 to 147) | 11986.8 (10389 to 13519) |
| Cameroon | Males | 5.4 (2.4 to 10.6) | 141.5 (79.1 to 235.2) | 3938.5 (2092.8 to 6036) |
| Cameroon | Females | 2.8 (1.3 to 5.3) | 108.5 (71 to 154.4) | 2090.3 (1236.4 to 3096.5) |
| Cameroon | Both sexes | 8.2 (4.3 to 15.3) | 250 (166.3 to 355.6) | 6028.9 (3634.4 to 8499.2) |
| Central African Republic | Males | 68.7 (44.4 to 101.5) | 43.4 (27.1 to 65.9) | 7404.2 (4694.5 to 10765.2) |
| Central African Republic | Females | 55.2 (34.4 to 92.8) | 25.1 (15.4 to 38.3) | 5707.8 (3400.3 to 8566.6) |
| Central African Republic | Both sexes | 123.9 (83.6 to 191.3) | 68.5 (46.6 to 97) | 13112 (9116 to 18021.4) |
| Chad | Males | 42.8 (24.9 to 71.9) | 89 (56 to 129.2) | 1536.1 (672.5 to 2637.1) |
| Chad | Females | 31.5 (17.8 to 53.5) | 70 (41.8 to 104.6) | 1483.8 (693.1 to 2420) |
| Chad | Both sexes | 74.2 (47.3 to 114.3) | 159 (110.8 to 218.5) | 3019.9 (1449.1 to 4563.6) |
| China | Males | 530.6 (272.6 to 949.3) | 3018 (1906.8 to 4264.2) | 4655.1 (2634.2 to 7397.9) |
| China | Females | 331.4 (173.2 to 594.2) | 1937.9 (1225.5 to 2575.6) | 3384.1 (1999.8 to 5414.8) |
| China | Both sexes | 862 (493.4 to 1393.5) | 4955.9 (3331.5 to 6442.1) | 8039.2 (5358.4 to 11976.9) |
| Colombia | Males | 10693.3 (5525.6 to 20807.7) | 139.3 (119.7 to 158.1) | 3732.1 (2227.3 to 5991.3) |
| Colombia | Females | 7953.3 (3789.7 to 15401.8) | 99.8 (84.9 to 123.1) | 2836.9 (1780.2 to 4482.3) |
| Colombia | Both sexes | 18646.6 (9934.3 to 34823.9) | 239.1 (216.1 to 263) | 6569 (4280.8 to 9049.6) |
| Comoros | Males | 0 (0 to 0) | 2.8 (1.4 to 4.5) | 2153.6 (1181 to 3558.9) |
| Comoros | Females | 0.1 (0 to 0.1) | 2.1 (1.3 to 3.3) | 2261.6 (1464 to 3437.8) |
| Comoros | Both sexes | 0.1 (0 to 0.1) | 4.9 (3 to 7.1) | 4415.2 (3000.8 to 6371.3) |
| Congo | Males | 252.2 (180.9 to 376.6) | 26.5 (17 to 39.8) | 156019.2 (98385.5 to 238168) |
| Congo | Females | 168.5 (112.5 to 259.2) | 16 (9.4 to 24.9) | 108693.8 (68883 to 167258.3) |
| Congo | Both sexes | 420.7 (301 to 617.3) | 42.5 (30.5 to 60.7) | 264713 (186552.3 to 379572.3) |
| Costa Rica | Males | 0.1 (0 to 0.2) | 10.6 (8.6 to 12.6) | 109.5 (52 to 187.3) |
| Costa Rica | Females | 0.3 (0.2 to 0.6) | 9.8 (7.7 to 12.5) | 94.6 (52.1 to 140.9) |
| Costa Rica | Both sexes | 0.4 (0.2 to 0.7) | 20.4 (17.6 to 23.2) | 204.1 (122.7 to 285.7) |
| C么te d'Ivoire | Males | 80.9 (47.3 to 138.2) | 102.8 (62.9 to 157) | 159.6 (87.7 to 262.4) |
| C么te d'Ivoire | Females | 47 (23.1 to 90.7) | 60.2 (39.7 to 86.1) | 88.7 (52.5 to 135.2) |
| C么te d'Ivoire | Both sexes | 127.9 (74 to 214.8) | 163 (116.7 to 225.9) | 248.3 (150.7 to 373.6) |
| Cuba | Males | 11.2 (5.7 to 19.8) | 15 (11.5 to 19) | 20715.1 (8668.9 to 37637) |
| Cuba | Females | 6.9 (4 to 11.8) | 8.6 (6.2 to 10.9) | 21241 (9773.8 to 35889.3) |
| Cuba | Both sexes | 18.1 (11.6 to 28.2) | 23.6 (19.2 to 27.7) | 41956.1 (24513.1 to 64245) |
| Democratic People's Republic of Korea | Males | 1.4 (0.6 to 2.8) | 54.1 (22.5 to 90.9) | 231 (121 to 379.1) |
| Democratic People's Republic of Korea | Females | 0.7 (0.3 to 1.6) | 51.4 (25.2 to 81.1) | 177.7 (109.7 to 275.3) |
| Democratic People's Republic of Korea | Both sexes | 2.2 (1.1 to 4.2) | 105.5 (55.8 to 156.4) | 408.6 (250.6 to 586) |
| Democratic Republic of the Congo | Males | 8.3 (5.5 to 12.7) | 491.7 (286.3 to 767.4) | 9402.8 (4645.4 to 15702.9) |
| Democratic Republic of the Congo | Females | 5.8 (3.5 to 9.8) | 285.2 (164.5 to 425.6) | 6468.2 (3451.5 to 10044.6) |
| Democratic Republic of the Congo | Both sexes | 14.1 (9 to 22.2) | 776.9 (519 to 1127.2) | 15871.1 (9432.8 to 24913.3) |
| Djibouti | Males | 3.2 (1.6 to 5.6) | 4.6 (2.3 to 7.9) | 1.6 (0.4 to 3.9) |
| Djibouti | Females | 2.1 (0.9 to 4.1) | 2.9 (1.7 to 4.7) | 15.2 (7.9 to 27) |
| Djibouti | Both sexes | 5.3 (2.5 to 9.6) | 7.5 (4.4 to 11.3) | 16.8 (8.7 to 29.6) |
| Dominica | Males | 1.1 (0.1 to 2.9) | 0.2 (0.1 to 0.3) | 5340.8 (3196.2 to 8278.2) |
| Dominica | Females | 1.6 (0.8 to 2.8) | 0.1 (0.1 to 0.1) | 5624.2 (3558.3 to 8742.6) |
| Dominica | Both sexes | 2.8 (1.2 to 5) | 0.3 (0.2 to 0.4) | 10965 (7336 to 16155.7) |
| Dominican Republic | Males | 12.8 (6 to 25.6) | 19.4 (11.1 to 32.1) | 40592.6 (23794.6 to 63189.2) |
| Dominican Republic | Females | 6.2 (3.2 to 11.2) | 13.9 (8.3 to 20.7) | 23293.1 (13343.1 to 34766.5) |
| Dominican Republic | Both sexes | 19 (9.3 to 34.2) | 33.3 (21 to 47.8) | 63885.7 (42803.8 to 92947.1) |
| Ecuador | Males | 6.4 (4 to 10.7) | 93.3 (78.9 to 107.8) | 2433.2 (1895.3 to 3095.2) |
| Ecuador | Females | 4.8 (2.8 to 8.6) | 75.5 (64.4 to 87.8) | 1965.6 (1505.9 to 2584) |
| Ecuador | Both sexes | 11.1 (7 to 18.7) | 168.8 (152.1 to 187) | 4398.8 (3615.3 to 5285) |
| Egypt | Males | 75.5 (46.1 to 124.3) | 317.8 (177.2 to 509) | 142.5 (100.2 to 198.3) |
| Egypt | Females | 57.5 (32.9 to 107.2) | 247.7 (147.1 to 384.4) | 116.2 (82.1 to 157) |
| Egypt | Both sexes | 133 (82 to 233.2) | 565.5 (382.3 to 812.5) | 258.8 (199.7 to 333.7) |
| El Salvador | Males | 5.2 (2.8 to 8.2) | 26.4 (15.1 to 37) | 26179.1 (14413.9 to 41793.9) |
| El Salvador | Females | 3.7 (2 to 6.3) | 22 (14.6 to 30) | 20540.7 (12130.4 to 31355.2) |
| El Salvador | Both sexes | 8.9 (5 to 14.9) | 48.3 (31.7 to 62) | 46719.8 (31601.2 to 67098.1) |
| Equatorial Guinea | Males | 16.7 (8.3 to 28.6) | 7.2 (4.1 to 11.7) | 51919.1 (31141.7 to 87983.8) |
| Equatorial Guinea | Females | 13.2 (5.8 to 26.9) | 2.7 (1.5 to 4.5) | 41444.3 (21952.6 to 66492.7) |
| Equatorial Guinea | Both sexes | 29.9 (15.2 to 55.8) | 9.9 (6.2 to 15.9) | 93363.4 (59490 to 136346.2) |
| Eritrea | Males | 9.2 (6.5 to 13.3) | 51.5 (28 to 81.8) | 9068.6 (3162 to 15755.6) |
| Eritrea | Females | 7.3 (4.4 to 11.9) | 34.3 (22 to 52.2) | 4493.3 (2701.3 to 6907.3) |
| Eritrea | Both sexes | 16.5 (11.7 to 24.8) | 85.8 (54 to 125.1) | 13561.9 (6410 to 21238) |
| Eswatini | Males | 678.6 (334.8 to 1066.4) | 3.4 (2 to 5.3) | 5805.2 (3623.3 to 9356.9) |
| Eswatini | Females | 546.5 (299.7 to 984.2) | 3.1 (2 to 4.6) | 4934.6 (3290 to 7419) |
| Eswatini | Both sexes | 1225.2 (721.9 to 2032.9) | 6.4 (4.3 to 9) | 10739.8 (7349.8 to 15790) |
| Ethiopia | Males | 0.1 (0.1 to 0.2) | 1758 (1029.7 to 2767.2) | 136.6 (79.6 to 230.4) |
| Ethiopia | Females | 0.2 (0.2 to 0.4) | 867.4 (510 to 1379.2) | 89.4 (48 to 149.9) |
| Ethiopia | Both sexes | 0.4 (0.3 to 0.6) | 2625.4 (1757.5 to 3834.3) | 225.9 (151.3 to 339.4) |
| Fiji | Males | 41.9 (24 to 68.8) | 0.7 (0.1 to 1.8) | 15570.7 (7597.5 to 27432.2) |
| Fiji | Females | 27.2 (14 to 54.1) | 0.8 (0.5 to 1.5) | 11130.1 (6271.6 to 18062.2) |
| Fiji | Both sexes | 69 (39.5 to 121.5) | 1.6 (0.7 to 2.9) | 26700.8 (16045.9 to 41876.2) |
| Gabon | Males | 154.3 (119.9 to 204.8) | 8.1 (5 to 12.6) | 2359.5 (1322.7 to 4010.3) |
| Gabon | Females | 130.2 (94.8 to 189.8) | 4.2 (2.5 to 6.7) | 1694 (933.1 to 2910.4) |
| Gabon | Both sexes | 284.5 (228.4 to 376.4) | 12.2 (8.7 to 16.9) | 4053.5 (2595.2 to 6244.3) |
| Gambia | Males | 0.2 (0.1 to 0.3) | 2.1 (1.3 to 3.4) | 13061.4 (5572.4 to 22078.6) |
| Gambia | Females | 0.2 (0.2 to 0.4) | 3.2 (2 to 5) | 16168.2 (7465.7 to 27703.7) |
| Gambia | Both sexes | 0.4 (0.3 to 0.6) | 5.3 (3.6 to 7.8) | 29229.6 (16341.2 to 43137.3) |
| Georgia | Males | 202.8 (138.7 to 306.3) | 2.5 (1.8 to 3.2) | 21621.1 (12548.9 to 33879.9) |
| Georgia | Females | 149.6 (88.4 to 264.5) | 2 (1.5 to 2.6) | 20131.2 (12338.9 to 32889.1) |
| Georgia | Both sexes | 352.3 (230.1 to 558.7) | 4.5 (3.6 to 5.4) | 41752.3 (28624.7 to 56938.3) |
| Ghana | Males | 61.5 (37.5 to 95) | 130.9 (74.2 to 230.7) | 21555.3 (14110.2 to 31584.8) |
| Ghana | Females | 47.7 (27.4 to 84.4) | 62.4 (40 to 99) | 11995.5 (7051.7 to 19240.3) |
| Ghana | Both sexes | 109.2 (66.8 to 170.5) | 193.3 (122.6 to 293.2) | 33550.8 (23399.8 to 46873.7) |
| Grenada | Males | 344.9 (236.8 to 522.2) | 0.1 (0 to 0.1) | 18028.2 (10821.1 to 29232.4) |
| Grenada | Females | 290 (167.1 to 521.4) | 0.1 (0.1 to 0.2) | 11027.3 (6724.5 to 18171.2) |
| Grenada | Both sexes | 634.8 (411.4 to 1017.3) | 0.2 (0.2 to 0.3) | 29055.5 (19826.7 to 41349.7) |
| Guatemala | Males | 90 (42 to 150.3) | 96.4 (77.5 to 121) | 143029.4 (83765.9 to 224849.6) |
| Guatemala | Females | 140 (80.2 to 217.9) | 76.5 (61 to 94.9) | 69267.1 (40658.7 to 110099.2) |
| Guatemala | Both sexes | 230 (150.2 to 351.3) | 172.9 (146.4 to 199.6) | 212296.5 (142078 to 309935.5) |
| Guinea | Males | 183.3 (139.4 to 252.7) | 17.8 (9.8 to 28.3) | 90961.8 (57072.9 to 139915.1) |
| Guinea | Females | 160.5 (110.8 to 242.4) | 35.3 (22.6 to 52.7) | 48515.6 (31437.7 to 71340.4) |
| Guinea | Both sexes | 343.8 (255.4 to 491.7) | 53 (34.9 to 74.2) | 139477.4 (98809.9 to 194603.6) |
| Guinea-Bissau | Males | 33.4 (17.5 to 62.2) | 8.3 (5.1 to 12.5) | 2398.7 (1592.5 to 3391.5) |
| Guinea-Bissau | Females | 26.5 (14.6 to 43.8) | 7.2 (4.5 to 10.8) | 1747.6 (1172.6 to 2535.4) |
| Guinea-Bissau | Both sexes | 59.9 (34.9 to 91.3) | 15.6 (10.7 to 21.6) | 4146.3 (2898.4 to 5538.4) |
| Haiti | Males | 33.1 (19.9 to 49.4) | 63.7 (29.1 to 106.3) | 16140.3 (8069.8 to 26690.8) |
| Haiti | Females | 29.5 (19.4 to 48.4) | 99.2 (57 to 149.7) | 12731.2 (6964.1 to 19569.4) |
| Haiti | Both sexes | 62.6 (42.5 to 88.8) | 162.9 (103.4 to 251) | 28871.5 (16832.2 to 43436.2) |
| Honduras | Males | 276.5 (131.7 to 475.3) | 26.4 (14.6 to 43.9) | 294.7 (223.2 to 380.8) |
| Honduras | Females | 212.5 (92.4 to 441.1) | 27.7 (18 to 42) | 191.9 (150 to 243.7) |
| Honduras | Both sexes | 489 (248.2 to 882.4) | 54.2 (36.6 to 78) | 486.7 (403.7 to 584.1) |
| India | Males | 1.5 (1.1 to 2.1) | 1914.8 (1195.4 to 2919.2) | 0.3 (0.1 to 0.7) |
| India | Females | 1.3 (0.9 to 2.1) | 1328.1 (842.8 to 2035.1) | 0.9 (0.5 to 1.5) |
| India | Both sexes | 2.9 (2.1 to 4.1) | 3242.8 (2275.5 to 4668.2) | 1.2 (0.6 to 1.9) |
| Indonesia | Males | 49.7 (27.9 to 77.1) | 974.8 (561.1 to 1569.8) | 2542.3 (1368 to 4093.6) |
| Indonesia | Females | 45.4 (27.8 to 73.4) | 637.6 (308.1 to 1049.8) | 1639.6 (927.3 to 2641.6) |
| Indonesia | Both sexes | 95.1 (64 to 151.4) | 1612.4 (942.2 to 2359.9) | 4181.9 (2696.3 to 6160.9) |
| Iran (Islamic Republic of) | Males | 1142.4 (898.8 to 1499.3) | 267.5 (155.5 to 419.6) | 14.5 (4.3 to 37) |
| Iran (Islamic Republic of) | Females | 901.3 (643.9 to 1395.9) | 243.3 (148.7 to 398.9) | 62 (34.3 to 105) |
| Iran (Islamic Republic of) | Both sexes | 2043.7 (1579.1 to 2830.4) | 510.8 (347.1 to 700.8) | 76.4 (42 to 133) |
| Iraq | Males | 0.3 (0.2 to 0.4) | 160.7 (85.4 to 255.1) | 44.3 (23.3 to 76.2) |
| Iraq | Females | 0.2 (0.1 to 0.3) | 124.8 (70.8 to 194.4) | 18.4 (10 to 30.1) |
| Iraq | Both sexes | 0.5 (0.3 to 0.8) | 285.5 (167.8 to 402.1) | 62.6 (35.5 to 97.9) |
| Jamaica | Males | 102.8 (60.5 to 161.2) | 5 (3.8 to 6.3) | 11429.5 (6971.8 to 17869.3) |
| Jamaica | Females | 109.6 (68.9 to 175.5) | 3.4 (2.5 to 4.3) | 7505.1 (4622.6 to 11742.4) |
| Jamaica | Both sexes | 212.3 (138.5 to 313.2) | 8.3 (6.8 to 9.9) | 18934.6 (13043.4 to 27391) |
| Jordan | Males | 2.1 (1 to 3.5) | 14.5 (5.7 to 26.1) | 396.9 (304.5 to 505.5) |
| Jordan | Females | 1.9 (1 to 3.1) | 16.5 (8.6 to 27.2) | 275.4 (207.2 to 349.3) |
| Jordan | Both sexes | 4 (2.2 to 6.5) | 31 (15.8 to 47.3) | 672.3 (545.9 to 805.4) |
| Kazakhstan | Males | 49.8 (18.4 to 103) | 29.8 (23.1 to 37.9) | 4897.4 (1734.6 to 9984.5) |
| Kazakhstan | Females | 68 (27.8 to 143.3) | 23.9 (18.2 to 31.3) | 7763.4 (4959.1 to 11730.9) |
| Kazakhstan | Both sexes | 117.8 (51.5 to 240) | 53.7 (44.4 to 64.2) | 12660.8 (7659.1 to 19764.9) |
| Kenya | Males | 46.3 (19.8 to 86.4) | 139.7 (86.8 to 214.6) | 0.5 (0 to 2) |
| Kenya | Females | 49.9 (20.7 to 106) | 91.5 (52.8 to 147.2) | 2.7 (1.5 to 4.6) |
| Kenya | Both sexes | 96.2 (46.7 to 174.9) | 231.2 (159.6 to 332.4) | 3.1 (1.6 to 5.8) |
| Kiribati | Males | 28.4 (17.9 to 44.5) | 0 (0 to 0) | 7699.3 (6192.4 to 9593.9) |
| Kiribati | Females | 27.4 (15.1 to 50.7) | 0.2 (0.1 to 0.3) | 6132.6 (4912.1 to 7580.4) |
| Kiribati | Both sexes | 55.8 (33.6 to 95.6) | 0.2 (0.1 to 0.3) | 13832 (11759.3 to 15947.3) |
| Kyrgyzstan | Males | 0.3 (0.2 to 0.4) | 11.8 (8.7 to 15.1) | 14.5 (10.2 to 21) |
| Kyrgyzstan | Females | 0.2 (0.1 to 0.2) | 5.9 (4.5 to 7.7) | 7.6 (5.2 to 10.7) |
| Kyrgyzstan | Both sexes | 0.4 (0.3 to 0.6) | 17.6 (14.2 to 21.7) | 22.1 (16.6 to 30) |
| Lao People's Democratic Republic | Males | 629.9 (496.4 to 856.8) | 28.9 (16.2 to 49.1) | 508.4 (262.7 to 874.5) |
| Lao People's Democratic Republic | Females | 577.8 (381.6 to 937) | 20.8 (11.4 to 35.6) | 325.7 (186.3 to 518.5) |
| Lao People's Democratic Republic | Both sexes | 1207.8 (899.3 to 1751.2) | 49.6 (31.6 to 76.5) | 834.1 (518.9 to 1230.1) |
| Lebanon | Males | 714.7 (338.2 to 1264.1) | 17 (7.4 to 28.9) | 9941.4 (8041.8 to 12018.9) |
| Lebanon | Females | 609.5 (316.8 to 1048.3) | 11.1 (6.2 to 17.8) | 6911.2 (5489.8 to 8327.5) |
| Lebanon | Both sexes | 1324.2 (759.3 to 2082.9) | 28.1 (16.2 to 41) | 16852.6 (14524.5 to 19332) |
| Lesotho | Males | 41.2 (21.6 to 64.5) | 3.4 (1.9 to 5.7) | 4277.5 (2352.5 to 6826) |
| Lesotho | Females | 44.8 (27.7 to 72.2) | 2.3 (1.4 to 3.5) | 2851.6 (1831.5 to 4351) |
| Lesotho | Both sexes | 86 (58 to 122) | 5.7 (3.8 to 8.4) | 7129.1 (4496.8 to 10392) |
| Liberia | Males | 3106 (1832.6 to 4860.9) | 17.7 (9.7 to 29.4) | 3611.7 (2239.4 to 5502.3) |
| Liberia | Females | 2234.5 (1350.5 to 3499.9) | 14.3 (9.1 to 22.2) | 2091.1 (1284.1 to 3182.5) |
| Liberia | Both sexes | 5340.4 (3610 to 7646.3) | 32 (20.6 to 45.6) | 5702.8 (3871.8 to 8067.4) |
| Libya | Males | 457.9 (237.8 to 882.3) | 18.6 (8.3 to 32) | 24568.2 (12513.8 to 33634.4) |
| Libya | Females | 346.7 (150.4 to 716.2) | 17.8 (8.2 to 28.7) | 18111.6 (10335.3 to 23834.4) |
| Libya | Both sexes | 804.6 (415.2 to 1493.6) | 36.4 (17.5 to 54.9) | 42679.8 (26910.1 to 55076.7) |
| Madagascar | Males | 61.6 (25.5 to 117.8) | 130.1 (64.1 to 207.8) | 31979.7 (16518.8 to 47855.8) |
| Madagascar | Females | 48.8 (19.7 to 104.4) | 85.4 (52.5 to 131.4) | 23847.8 (14328 to 35654.6) |
| Madagascar | Both sexes | 110.4 (49.6 to 211.4) | 215.5 (130.3 to 317.9) | 55827.5 (35679 to 76741.3) |
| Malawi | Males | 33.9 (15.3 to 68.5) | 25.8 (12 to 43.5) | 318.4 (159.2 to 523.2) |
| Malawi | Females | 26.3 (12.4 to 51.9) | 33 (19 to 52.6) | 574.2 (358.9 to 866.1) |
| Malawi | Both sexes | 60.2 (29.3 to 113.1) | 58.8 (34.9 to 92) | 892.6 (581.5 to 1285.4) |
| Malaysia | Males | 411.5 (203.7 to 710.6) | 42.3 (23.2 to 80.8) | 176.5 (105.9 to 282.2) |
| Malaysia | Females | 374.1 (169.5 to 812.9) | 28.5 (16.1 to 46) | 259 (161.1 to 404) |
| Malaysia | Both sexes | 785.7 (426 to 1489.2) | 70.8 (44.6 to 114.8) | 435.5 (293.4 to 630.5) |
| Maldives | Males | 365.6 (152.7 to 683.5) | 0.5 (0.3 to 0.9) | 120.9 (68.3 to 241.8) |
| Maldives | Females | 388.9 (179.2 to 662.9) | 0.2 (0.1 to 0.3) | 58.3 (36.7 to 98.1) |
| Maldives | Both sexes | 754.5 (432.5 to 1173.3) | 0.8 (0.4 to 1.2) | 179.2 (114 to 300.6) |
| Mali | Males | 718.9 (423.8 to 1104.5) | 105.7 (37.7 to 183.2) | 13088.4 (6957.3 to 20799.5) |
| Mali | Females | 414 (234.9 to 651.9) | 54.5 (33 to 83.6) | 10332.5 (5899 to 16168.1) |
| Mali | Both sexes | 1132.9 (763.2 to 1619.5) | 160.2 (77.2 to 249.7) | 23420.9 (13726.8 to 32685.1) |
| Marshall Islands | Males | 388.7 (246.1 to 559) | 0 (0 to 0) | 10779.9 (5363.7 to 17198.6) |
| Marshall Islands | Females | 217.6 (129.3 to 355) | 0 (0 to 0.1) | 6983.2 (4305 to 10747.5) |
| Marshall Islands | Both sexes | 606.3 (415.9 to 826.7) | 0 (0 to 0.1) | 17763 (10717 to 26252.6) |
| Mauritania | Males | 115.6 (52.3 to 217.1) | 14.3 (8.2 to 23.2) | 358.7 (204.1 to 609.3) |
| Mauritania | Females | 86.7 (36.1 to 193.6) | 12.3 (8.1 to 18.7) | 201.2 (114.2 to 309.6) |
| Mauritania | Both sexes | 202.2 (97.2 to 369.3) | 26.6 (18.1 to 36.6) | 559.9 (365.5 to 888) |
| Mauritius | Males | 283.8 (127.5 to 478.9) | 1 (0.8 to 1.2) | 2749.6 (1840.7 to 3875.7) |
| Mauritius | Females | 309.7 (146.1 to 548.4) | 0.8 (0.7 to 1.1) | 1304.9 (684.5 to 2086.4) |
| Mauritius | Both sexes | 593.5 (334.1 to 891.5) | 1.8 (1.5 to 2.1) | 4054.5 (2673.2 to 5365.8) |
| Mexico | Males | 964.1 (564.2 to 1616.7) | 639.7 (571.4 to 725.6) | 2194.2 (1406.1 to 3298.6) |
| Mexico | Females | 790 (423.4 to 1286.2) | 452 (395.2 to 513.1) | 1307.1 (766.3 to 2021.7) |
| Mexico | Both sexes | 1754 (1110.8 to 2577.7) | 1091.7 (998 to 1194.3) | 3501.3 (2496.1 to 5010.3) |
| Micronesia (Federated States of) | Males | 2.7 (1.6 to 4.7) | 0 (0 to 0) | 27227.3 (24231.4 to 30889) |
| Micronesia (Federated States of) | Females | 1.9 (1.1 to 3.5) | 0 (0 to 0) | 21989.7 (19049.3 to 25061.1) |
| Micronesia (Federated States of) | Both sexes | 4.6 (2.9 to 7.4) | 0 (0 to 0.1) | 49217 (44542.2 to 54734.1) |
| Mongolia | Males | 38.4 (17 to 69.6) | 6.2 (3.2 to 10.6) | 273.9 (155.6 to 455.9) |
| Mongolia | Females | 36.2 (18.8 to 59.3) | 4 (2.3 to 6.3) | 186.2 (116.8 to 288.9) |
| Mongolia | Both sexes | 74.6 (41.7 to 121.8) | 10.2 (6.3 to 14.9) | 460.2 (306.6 to 670.9) |
| Montenegro | Males | 2551.1 (1487.6 to 4148.1) | 0.8 (0.5 to 1.1) | 666.4 (415.6 to 1046.1) |
| Montenegro | Females | 1217.9 (748.5 to 1999.6) | 0.6 (0.4 to 0.8) | 339.8 (207.9 to 548.7) |
| Montenegro | Both sexes | 3769 (2551.4 to 5792.1) | 1.4 (1 to 1.8) | 1006.2 (712.7 to 1407.1) |
| Morocco | Males | 12.9 (7.9 to 19.9) | 20.5 (9.8 to 38.8) | 247231.4 (155193.5 to 350359.4) |
| Morocco | Females | 6.6 (4 to 10.7) | 18.6 (10.3 to 30) | 160404.6 (103356.1 to 211403.8) |
| Morocco | Both sexes | 19.5 (13 to 27.7) | 39.1 (23.3 to 60.5) | 407636 (273216.5 to 527135.6) |
| Mozambique | Males | 4.2 (2.2 to 6.9) | 57.4 (20.4 to 118.2) | 203.9 (115.3 to 326.8) |
| Mozambique | Females | 3.2 (2 to 5.1) | 91.2 (58.3 to 138.1) | 159.1 (89.3 to 253.6) |
| Mozambique | Both sexes | 7.4 (4.5 to 10.7) | 148.5 (89.8 to 232.3) | 363 (229.5 to 523.8) |
| Myanmar | Males | 39.8 (25.1 to 60.9) | 222.8 (125.1 to 375.8) | 1584 (958.8 to 2297.1) |
| Myanmar | Females | 23.7 (14.4 to 37.6) | 161.9 (90 to 257) | 1332 (888.9 to 1813.5) |
| Myanmar | Both sexes | 63.5 (43.9 to 93.7) | 384.7 (250.5 to 572) | 2915.9 (1982.2 to 3812.6) |
| Namibia | Males | 937.6 (444.8 to 1790.4) | 8 (4.3 to 12.6) | 5.6 (4 to 7.4) |
| Namibia | Females | 1066.2 (406.4 to 2298.7) | 8.4 (4.9 to 12.9) | 11.4 (8.8 to 14.8) |
| Namibia | Both sexes | 2003.8 (889.9 to 3844.3) | 16.3 (10.3 to 23.5) | 17 (13.9 to 20.5) |
| Nepal | Males | 158.8 (77.6 to 266.8) | 66 (40.5 to 115.2) | 1214.5 (936.9 to 1546.9) |
| Nepal | Females | 109.5 (59.9 to 172.1) | 43.6 (25 to 69.2) | 697.2 (509.8 to 880.3) |
| Nepal | Both sexes | 268.3 (158.8 to 417.5) | 109.6 (72.9 to 169.8) | 1911.7 (1558.4 to 2262.3) |
| Nicaragua | Males | 95.1 (42.6 to 176.8) | 29.7 (19.8 to 42.1) | 60.5 (7.1 to 148.8) |
| Nicaragua | Females | 70.8 (35 to 126.3) | 21.7 (14.7 to 31.5) | 74.2 (40.8 to 128.7) |
| Nicaragua | Both sexes | 165.9 (89 to 294) | 51.5 (35.7 to 68.3) | 134.7 (58.5 to 244.3) |
| Niger | Males | 189.3 (95.1 to 302) | 192.3 (95.9 to 317.9) | 14297.1 (6308.8 to 23677.9) |
| Niger | Females | 122.7 (77.5 to 190.8) | 154.7 (84.9 to 239.1) | 14640.7 (6613.5 to 24132.7) |
| Niger | Both sexes | 312 (189.4 to 473.3) | 347 (203.4 to 520.1) | 28937.8 (16308.8 to 42348.9) |
| Nigeria | Males | 122 (56.4 to 213.6) | 1084.3 (684.3 to 1665.3) | 11211.1 (9589.2 to 12742.3) |
| Nigeria | Females | 91.7 (56.5 to 140.6) | 587.6 (382.4 to 863.2) | 8172.9 (6898.6 to 9949.5) |
| Nigeria | Both sexes | 213.8 (119.9 to 326.8) | 1671.9 (1184 to 2325.4) | 19384 (17440.2 to 21265) |
| North Macedonia | Males | 692.1 (396.1 to 1096.8) | 1.8 (1.2 to 2.4) | 10713.6 (6025.8 to 18981.5) |
| North Macedonia | Females | 422.7 (247.1 to 752.2) | 1.4 (1 to 1.9) | 4988.2 (3182.3 to 7865.8) |
| North Macedonia | Both sexes | 1114.8 (700.7 to 1822.2) | 3.2 (2.5 to 4.1) | 15701.9 (9874.7 to 23934.7) |
| Pakistan | Males | 86.9 (30.7 to 172.4) | 627 (376.9 to 1061.7) | 0.7 (0 to 2.9) |
| Pakistan | Females | 139.3 (88.6 to 217.3) | 499.3 (262.9 to 794) | 0.9 (0 to 3.6) |
| Pakistan | Both sexes | 226.3 (137.1 to 345) | 1126.3 (713.1 to 1647) | 1.6 (0.1 to 6) |
| Palestine | Males | 100 (59.7 to 169.4) | 12.2 (6.9 to 20.4) | 194.4 (144.4 to 256.5) |
| Palestine | Females | 67.3 (40.4 to 108.4) | 8.4 (5.2 to 12.5) | 158.4 (120.7 to 209.2) |
| Palestine | Both sexes | 167.2 (111.9 to 264.9) | 20.6 (14 to 30.6) | 352.8 (286.1 to 425.9) |
| Papua New Guinea | Males | 11.5 (6.4 to 18.8) | 6.7 (1.6 to 20) | 651.6 (351.9 to 1037.3) |
| Papua New Guinea | Females | 4.4 (2.5 to 7.8) | 16.2 (8.4 to 30.5) | 681.9 (401.7 to 1052.4) |
| Papua New Guinea | Both sexes | 15.9 (9.2 to 24.8) | 22.9 (11.8 to 41.5) | 1333.6 (845.7 to 1923.6) |
| Paraguay | Males | 74.4 (42.7 to 119.8) | 19.7 (11.9 to 28.7) | 77.1 (59.7 to 94.5) |
| Paraguay | Females | 50.1 (32.7 to 79.1) | 16.5 (11 to 22.3) | 67.4 (52.9 to 84.9) |
| Paraguay | Both sexes | 124.5 (80.5 to 181.8) | 36.2 (24.4 to 47.8) | 144.4 (121.9 to 166) |
| Peru | Males | 61 (38.3 to 93.3) | 302.2 (153.9 to 413) | 5377.6 (3311.1 to 9356.3) |
| Peru | Females | 35.6 (21.9 to 55) | 221.6 (127.5 to 291.1) | 3536.9 (2035.5 to 5598.3) |
| Peru | Both sexes | 96.7 (66.7 to 138.6) | 523.8 (328.6 to 675.2) | 8914.4 (5936.6 to 13874.6) |
| Philippines | Males | 6 (3.2 to 10) | 392 (203.7 to 589.4) | 6190.2 (3757.2 to 9728.8) |
| Philippines | Females | 10.9 (6.8 to 16.4) | 288.4 (171.4 to 429.7) | 4158.5 (2665.3 to 6112) |
| Philippines | Both sexes | 16.9 (11 to 23.7) | 680.4 (435.3 to 933.6) | 10348.7 (7278 to 13833.5) |
| Republic of Moldova | Males | 131.6 (76.9 to 213.6) | 2.4 (1.8 to 3.1) | 1198.3 (467.2 to 2162) |
| Republic of Moldova | Females | 108.7 (68.3 to 162.3) | 1.9 (1.5 to 2.4) | 1384 (715.5 to 2275.8) |
| Republic of Moldova | Both sexes | 240.2 (154.7 to 359.2) | 4.3 (3.5 to 5.1) | 2582.3 (1330.3 to 3972.6) |
| Rwanda | Males | 293.3 (142.8 to 525.6) | 67.3 (33 to 109.1) | 4622.5 (2322.9 to 7796.7) |
| Rwanda | Females | 211.7 (120.6 to 351.9) | 51.3 (31.6 to 79.6) | 3474.1 (2078.4 to 5250.8) |
| Rwanda | Both sexes | 505 (294 to 765.9) | 118.6 (68.5 to 178.5) | 8096.6 (4823.2 to 11938.5) |
| Saint Lucia | Males | 127.2 (79.9 to 185.8) | 0.2 (0.1 to 0.2) | 4.7 (1.2 to 12.3) |
| Saint Lucia | Females | 96.7 (58.6 to 144) | 0.1 (0.1 to 0.1) | 17 (8.7 to 28.8) |
| Saint Lucia | Both sexes | 223.9 (157.6 to 307.2) | 0.2 (0.2 to 0.3) | 21.6 (11 to 40.1) |
| Saint Vincent and the Grenadines | Males | 211.5 (126.2 to 338.7) | 0.1 (0.1 to 0.2) | 11778.9 (6590.9 to 19569.2) |
| Saint Vincent and the Grenadines | Females | 132.6 (79.4 to 219.8) | 0.1 (0.1 to 0.2) | 8805.9 (5758.6 to 12591.8) |
| Saint Vincent and the Grenadines | Both sexes | 344.1 (226.3 to 508) | 0.3 (0.2 to 0.3) | 20584.9 (13614 to 29350.9) |
| Samoa | Males | 7 (3.6 to 11.7) | 0 (0 to 0) | 51029.2 (45684.3 to 57700.8) |
| Samoa | Females | 4.3 (2.5 to 6.9) | 0 (0 to 0) | 36531.8 (31979.6 to 41518.6) |
| Samoa | Both sexes | 11.3 (6.7 to 16.8) | 0 (0 to 0.1) | 87561 (79937.5 to 95384.6) |
| Sao Tome and Principe | Males | 23.1 (13.4 to 36.1) | 0.1 (0.1 to 0.2) | 1378.7 (1030.1 to 1795.5) |
| Sao Tome and Principe | Females | 18.7 (11.7 to 28.8) | 0.3 (0.2 to 0.5) | 785.3 (591.4 to 1051.4) |
| Sao Tome and Principe | Both sexes | 41.8 (27.6 to 61.4) | 0.4 (0.3 to 0.6) | 2164.1 (1780 to 2716.1) |
| Senegal | Males | 64 (38.2 to 100.9) | 75 (45.3 to 117.2) | 10.6 (7.7 to 14.3) |
| Senegal | Females | 50 (30.4 to 79.5) | 51.4 (33.1 to 75) | 11.1 (8.5 to 14) |
| Senegal | Both sexes | 114 (77 to 161.7) | 126.3 (89 to 168.1) | 21.7 (18 to 26.5) |
| Serbia | Males | 104 (63.6 to 168.4) | 4.5 (2.5 to 7.7) | 79178.2 (45642.2 to 127520.1) |
| Serbia | Females | 86.3 (56.4 to 128.5) | 2.5 (1.4 to 3.9) | 51929 (25165 to 84705.2) |
| Serbia | Both sexes | 190.3 (130.4 to 276.6) | 7 (4.5 to 11.1) | 131107.2 (76426.7 to 191721.2) |
| Sierra Leone | Males | 1.9 (1.3 to 2.9) | 29.6 (15.3 to 47.9) | 2100.3 (1206.6 to 2992.5) |
| Sierra Leone | Females | 1.8 (1.1 to 3) | 23.8 (15.1 to 35) | 1768.2 (1186.7 to 2402.2) |
| Sierra Leone | Both sexes | 3.7 (2.6 to 5.6) | 53.4 (34.8 to 78.1) | 3868.4 (2558.6 to 4935.1) |
| Solomon Islands | Males | 3.2 (1.9 to 5.1) | 0.2 (0 to 0.5) | 8512.3 (5231.4 to 12966.7) |
| Solomon Islands | Females | 4.6 (2.8 to 7.2) | 0.7 (0.4 to 1.2) | 4894.9 (3230.5 to 6993.2) |
| Solomon Islands | Both sexes | 7.8 (5.2 to 11.3) | 0.9 (0.5 to 1.6) | 13407.2 (9612 to 18541.3) |
| Somalia | Males | 11.9 (6.6 to 18.7) | 112.4 (55.6 to 186.5) | 1389 (606 to 2357.4) |
| Somalia | Females | 12.7 (7.3 to 20.3) | 77.8 (41.4 to 120.5) | 925.5 (510.6 to 1453.3) |
| Somalia | Both sexes | 24.6 (15.8 to 35.3) | 190.3 (113.4 to 296.4) | 2314.5 (1347.6 to 3362.4) |
| South Africa | Males | 11.9 (7.2 to 18.2) | 83.7 (47.5 to 125.3) | 995.6 (564.1 to 1672.6) |
| South Africa | Females | 9.9 (6.3 to 14.5) | 65.8 (42.2 to 98.2) | 696.4 (423.8 to 1032.2) |
| South Africa | Both sexes | 21.7 (15.1 to 30.1) | 149.5 (100.5 to 203.7) | 1692 (1144.9 to 2490.4) |
| South Sudan | Males | 250.2 (133.4 to 400.1) | 55.6 (27.6 to 94) | 7071.9 (3244.8 to 12412.9) |
| South Sudan | Females | 76.6 (46.4 to 116) | 42.3 (25.2 to 64.2) | 5297.7 (3101.9 to 8008.7) |
| South Sudan | Both sexes | 326.8 (194 to 484.3) | 97.9 (58.3 to 144.3) | 12369.6 (6867 to 18566.6) |
| Sri Lanka | Males | 2.8 (1.4 to 6) | 34.4 (23.1 to 48.6) | 271.2 (160.6 to 423.2) |
| Sri Lanka | Females | 1.3 (0.7 to 2.4) | 15.8 (8 to 25.5) | 247.4 (160.3 to 372) |
| Sri Lanka | Both sexes | 4.1 (2.3 to 7.9) | 50.2 (32.5 to 66.4) | 518.5 (346.7 to 721.7) |
| Sudan | Males | 210.8 (128.6 to 328.7) | 159.4 (68.1 to 270.2) | 1487.2 (813.6 to 2383.6) |
| Sudan | Females | 138.4 (79.7 to 220) | 200.1 (92.7 to 343) | 2842.8 (1807.9 to 4232.4) |
| Sudan | Both sexes | 349.2 (228.6 to 521.2) | 359.5 (200.5 to 530.8) | 4330 (2840 to 6026.8) |
| Suriname | Males | 44.9 (24.4 to 74.1) | 1.4 (0.6 to 2.3) | 7572.4 (6395.6 to 8738.9) |
| Suriname | Females | 34.1 (21.6 to 51.6) | 1.2 (0.6 to 1.7) | 6150.8 (5226.3 to 7136.5) |
| Suriname | Both sexes | 79 (50.9 to 115.8) | 2.5 (1.5 to 3.5) | 13723.2 (12421.5 to 15289.3) |
| Syrian Arab Republic | Males | 101.7 (50.1 to 166.5) | 41.4 (19.2 to 70.6) | 1584 (906.8 to 2606) |
| Syrian Arab Republic | Females | 77.5 (47.5 to 122.8) | 27.1 (14.7 to 40.6) | 1158.6 (699.5 to 1716.9) |
| Syrian Arab Republic | Both sexes | 179.1 (104.1 to 262.6) | 68.6 (38.7 to 104.1) | 2742.6 (1714 to 3943.2) |
| Tajikistan | Males | 167.8 (55 to 285.5) | 29 (16 to 49.2) | 590.9 (337.9 to 959.7) |
| Tajikistan | Females | 80.1 (48.6 to 123.5) | 20.2 (11.9 to 30.7) | 220.8 (118.8 to 375.4) |
| Tajikistan | Both sexes | 247.9 (118.5 to 391.7) | 49.1 (32.5 to 77.9) | 811.8 (504.4 to 1314.1) |
| Thailand | Males | 1713.7 (1035 to 2622.8) | 85.5 (40.7 to 142.7) | 1658.1 (781.1 to 3156.1) |
| Thailand | Females | 907.4 (568.3 to 1350.6) | 49 (27.3 to 71.6) | 1502.8 (826.9 to 2393.8) |
| Thailand | Both sexes | 2621.1 (1811.6 to 3598.1) | 134.5 (75.3 to 203) | 3160.9 (1886.9 to 4866.2) |
| Timor-Leste | Males | 38.5 (17.8 to 65.7) | 7.9 (4.4 to 12.9) | 651.1 (360.1 to 1060.3) |
| Timor-Leste | Females | 47.7 (28 to 78.6) | 6 (3.4 to 9.5) | 489.9 (282.1 to 787) |
| Timor-Leste | Both sexes | 86.2 (50.4 to 136.3) | 13.8 (9.2 to 19.6) | 1141.1 (752.9 to 1612) |
| Togo | Males | 4.7 (2.7 to 7.2) | 25.5 (13.6 to 39.3) | 11432.3 (7142.2 to 17580.6) |
| Togo | Females | 4.3 (2.8 to 6.6) | 21.9 (13.6 to 32.9) | 7318.1 (4275.8 to 11737.7) |
| Togo | Both sexes | 9 (6.1 to 12.6) | 47.4 (31.8 to 65) | 18750.5 (12935 to 26950.1) |
| Tonga | Males | 26.3 (14.4 to 41.7) | 0 (0 to 0.1) | 13854.9 (7339.7 to 22544.3) |
| Tonga | Females | 48.3 (29.8 to 72.9) | 0 (0 to 0.1) | 4171.9 (2531 to 6380.1) |
| Tonga | Both sexes | 74.6 (48.9 to 105.4) | 0.1 (0 to 0.1) | 18026.8 (10588.7 to 26902.4) |
| Tunisia | Males | 4.6 (2.6 to 7.5) | 30.5 (16.4 to 48.5) | 0.9 (0.3 to 2.2) |
| Tunisia | Females | 3.2 (2 to 4.9) | 19.4 (11.1 to 31.2) | 2.9 (1.4 to 5.2) |
| Tunisia | Both sexes | 7.7 (5.2 to 11.4) | 49.9 (32 to 72.5) | 3.8 (2 to 6.8) |
| T眉rkiye | Males | 80.5 (41.1 to 130.1) | 146.1 (75.8 to 221.4) | 2122.4 (1122.4 to 3278.7) |
| T眉rkiye | Females | 41.9 (26.4 to 66.6) | 90 (55.5 to 134.2) | 1771.7 (1097.6 to 2668.2) |
| T眉rkiye | Both sexes | 122.4 (72.5 to 178.8) | 236.1 (145.4 to 330.7) | 3894.1 (2620.7 to 5368.9) |
| Turkmenistan | Males | 112.7 (68 to 176.2) | 17 (12.7 to 22.1) | 959.4 (717.9 to 1232.7) |
| Turkmenistan | Females | 74.5 (46 to 112.5) | 9.7 (7.3 to 13) | 479.8 (362.9 to 624.5) |
| Turkmenistan | Both sexes | 187.2 (128.1 to 259.1) | 26.7 (21.9 to 33.6) | 1439.2 (1160.7 to 1775.4) |
| Tuvalu | Males | 287.5 (145.7 to 476.2) | 0 (0 to 0) | 2.4 (0.6 to 5.9) |
| Tuvalu | Females | 222.9 (124 to 344.7) | 0 (0 to 0) | 3 (1.5 to 5.5) |
| Tuvalu | Both sexes | 510.4 (295.8 to 776.3) | 0 (0 to 0) | 5.4 (2.5 to 10.1) |
| Uganda | Males | 154.2 (91.4 to 240.9) | 186.2 (91.2 to 327.6) | 32960.6 (18926.2 to 50970.1) |
| Uganda | Females | 87.4 (56.7 to 124.1) | 134.1 (75.7 to 217) | 19630.9 (11570.3 to 32012.3) |
| Uganda | Both sexes | 241.6 (168.9 to 337.2) | 320.3 (191.4 to 503.3) | 52591.5 (34609.6 to 77053.1) |
| Ukraine | Males | 216.7 (117.3 to 360.7) | 27.7 (22.4 to 34.4) | 6809 (3820.2 to 10207.9) |
| Ukraine | Females | 157.7 (97.6 to 230.7) | 20.7 (15.7 to 27.3) | 5404.9 (3464.3 to 8099.8) |
| Ukraine | Both sexes | 374.4 (243.1 to 536.4) | 48.4 (40.6 to 58.9) | 12214 (8214.9 to 16654.5) |
| United Republic of Tanzania | Males | 196.4 (108.5 to 358.4) | 167.6 (87.6 to 272.1) | 384.9 (191.2 to 653.5) |
| United Republic of Tanzania | Females | 88.4 (56.1 to 138.6) | 50.4 (30.6 to 77.3) | 236.6 (137.7 to 386.4) |
| United Republic of Tanzania | Both sexes | 284.8 (183.8 to 443.2) | 218 (127 to 325.9) | 621.5 (366.3 to 932.6) |
| Uzbekistan | Males | 26.6 (14.4 to 43.1) | 119.2 (97 to 144.3) | 4427.6 (2221.7 to 7196.3) |
| Uzbekistan | Females | 20.5 (12.6 to 31.7) | 83 (66 to 99.5) | 2308.6 (1454.9 to 3664.8) |
| Uzbekistan | Both sexes | 47.1 (29.9 to 68.1) | 202.2 (174.4 to 232.2) | 6736.2 (3989.9 to 9770.9) |
| Vanuatu | Males | 79.2 (41.5 to 135) | 0.1 (0 to 0.1) | 3404.7 (1870.8 to 6425.2) |
| Vanuatu | Females | 59.3 (35.9 to 90.8) | 0.2 (0.1 to 0.3) | 2321.4 (1312 to 3736.1) |
| Vanuatu | Both sexes | 138.5 (83 to 204.2) | 0.3 (0.1 to 0.5) | 5726.1 (3625.3 to 9266.3) |
| Viet Nam | Males | 38.3 (20.2 to 60.9) | 219.5 (131.6 to 355.4) | 2161.3 (1002.5 to 3665.4) |
| Viet Nam | Females | 31.1 (19.6 to 47.5) | 135.9 (82.3 to 226.2) | 2705.2 (1563.9 to 4310.3) |
| Viet Nam | Both sexes | 69.4 (46.5 to 97.6) | 355.4 (240.4 to 502.8) | 4866.6 (2903.7 to 7550.2) |
| Yemen | Males | 0.2 (0.1 to 0.4) | 175.6 (77.2 to 291.7) | 1464.2 (794.2 to 2446.2) |
| Yemen | Females | 0.5 (0.3 to 0.7) | 177.9 (81 to 292.4) | 1152.4 (728.5 to 1787.1) |
| Yemen | Both sexes | 0.7 (0.5 to 1) | 353.5 (198.9 to 516.9) | 2616.6 (1688 to 3745.2) |
| Zambia | Males | 0 (0 to 0) | 52.9 (26.3 to 86.1) | 1180.1 (673.6 to 1923.1) |
| Zambia | Females | 0 (0 to 0) | 28 (17.6 to 44.2) | 986.6 (641.7 to 1495.5) |
| Zambia | Both sexes | 0 (0 to 0) | 81 (47.8 to 117) | 2166.6 (1479.4 to 2985.5) |
| Zimbabwe | Males | 291.4 (115.6 to 529.1) | 45.8 (27.4 to 73.2) | 3283.1 (1528.9 to 5591.4) |
| Zimbabwe | Females | 362.2 (157.4 to 647.4) | 34.5 (21.5 to 54.3) | 2174.1 (1183.4 to 3276) |
| Zimbabwe | Both sexes | 653.6 (345.5 to 1052.6) | 80.3 (52.2 to 110.6) | 5457.1 (3114.9 to 8308.5) |
