# Supplementary material for: Burden of acute lymphoblastic leukemia in children and adolescents in low- and middle-income countries from 1990 to 2023 and projections to 2050: A systematic analysis from the global burden of disease study 2023
Source: PLoS One. 2026 Jun 2;21(6):e0350223. doi: 10.1371/journal.pone.0350223 (PMC13229300; doi:10.1371/journal.pone.0350223)
Supplement: S2 Table — (DOCX) [file pone.0350223.s002.docx]

| S2 Table. Age-standardized incidence, mortality and DALYs rate of acute lymphoblastic leukemia in 2023 | | | | |
| --- | --- | --- | --- | --- |
| **Locations** | **Sex** | **ASIR and 95%UI** | **ASMR and 95%UI** | **ASDR and 95%UI** |
| World Bank Upper Middle Income | Male | 4.72 (2.8 to 8.1 ) | 1.75 (1.2 to 2.2) | 141.88 (100.5 to 180.7) |
| World Bank Upper Middle Income | Female | 3.99 (2.18 to 7.0) | 1.3 (1.0 to 1.6) | 106.91 (81.7 to 127.3) |
| World Bank Upper Middle Income | Both | 4.37 (2.7 to 7.5) | 1.53(1.2 to 1.8) | 125.11 (94.1 to 150.2) |
| World Bank Lower Middle Income | Male | 1.87 (1.19 to 2.84) | 1.12 (0.7 to 1.7) | 92.2 (60.3 to 140.7) |
| World Bank Lower Middle Income | Female | 1.37（0.9 to 2.1) | 0.8 (0.5 to1.2) | 65.57 (43.4 to 94.8) |
| World Bank Lower Middle Income | Both | 1.63 (1.2 to 2.3) | 0.96 (0.7 to 1.3) | 79.24 (57.1 to 108.0) |
| World Bank Low Income | Male | 3.53 (2.3 to 5.5) | 2.36 (1.5 to 3.7) | 193.96 (125.4 to 302.7) |
| World Bank Low Income | Female | 2.49 (1.6 to 3.9) | 1.65 (1.1 to 2.5) | 134.48 (88.8 to 201.2) |
| World Bank Low Income | Both | 3.02(2.1 to 4.2) | 2.01 (1.4 to2.8) | 164.65 (115.4 to 233.0) |
| Türkiye | Males | 0 (0 to 0.1) | 1.1 (0.6 to 1.7) | 53.6 (40.6 to 69.4) |
| Türkiye | Females | 0 (0 to 0.1) | 0.8 (0.5 to 1.1) | 45.6 (35.5 to 58.7) |
| Türkiye | Both sexes | 0 (0 to 0.1) | 0.9 (0.6 to 1.3) | 49.8 (40.7 to 60) |
| Afghanistan | Males | 3.6 (2.1 to 5.8) | 2.4 (1 to 4.3) | 77 (54 to 109.8) |
| Afghanistan | Females | 2.9 (1.7 to 4.8) | 2.6 (1.2 to 4.4) | 62.3 (43.1 to 87.1) |
| Afghanistan | Both sexes | 3.3 (2.2 to 4.8) | 2.5 (1.5 to 3.8) | 69.9 (52.5 to 92.9) |
| Albania | Males | 3.1 (1.5 to 5.7) | 0.9 (0.5 to 1.4) | 175.4 (80.6 to 294.9) |
| Albania | Females | 2.3 (1.1 to 4.8) | 0.7 (0.4 to 1.2) | 277.5 (160.3 to 418.2) |
| Albania | Both sexes | 2.7 (1.4 to 4.8) | 0.8 (0.5 to 1.2) | 225.8 (143.5 to 348.9) |
| Algeria | Males | 3.3 (1.9 to 5.3) | 1.2 (0.7 to 1.9) | 115.1 (93.9 to 137.7) |
| Algeria | Females | 2.4 (1.2 to 3.9) | 0.8 (0.5 to 1.3) | 108.2 (85.4 to 137.2) |
| Algeria | Both sexes | 2.8 (1.7 to 4.2) | 1 (0.7 to 1.5) | 111.7 (95.8 to 127.3) |
| Angola | Males | 1 (0.7 to 1.4) | 2.6 (1.7 to 3.7) | 21.5 (12.7 to 35.3) |
| Angola | Females | 0.9 (0.6 to 1.4) | 1.4 (0.8 to 2.3) | 49.8 (31.4 to 78.1) |
| Angola | Both sexes | 0.9 (0.6 to 1.4) | 2 (1.4 to 2.8) | 35.5 (24.6 to 50.7) |
| Argentina | Males | 2.7 (1.4 to 4) | 1.3 (1 to 1.5) | 19.8 (4.8 to 59.6) |
| Argentina | Females | 2.3 (1.3 to 3.7) | 0.9 (0.7 to 1) | 55.3 (28.9 to 101.2) |
| Argentina | Both sexes | 2.5 (1.6 to 3.5) | 1.1 (0.9 to 1.2) | 36.6 (19 to 66.1) |
| Armenia | Males | 2.5 (1.8 to 3.5) | 0.9 (0.7 to 1.2) | 59 (42.8 to 77.7) |
| Armenia | Females | 1.5 (1 to 2.1) | 0.7 (0.5 to 0.8) | 36.3 (26.7 to 47.6) |
| Armenia | Both sexes | 2 (1.6 to 2.8) | 0.8 (0.7 to 1) | 47.9 (38.4 to 59.3) |
| Azerbaijan | Males | 0.2 (0 to 0.4) | 2.9 (1.6 to 4.5) | 92.8 (48 to 139.6) |
| Azerbaijan | Females | 0.6 (0.3 to 1.2) | 1.8 (1 to 2.6) | 61.4 (39 to 90.8) |
| Azerbaijan | Both sexes | 0.4 (0.2 to 0.7) | 2.4 (1.4 to 3.3) | 77.5 (48.5 to 106.7) |
| Bangladesh | Males | 0.2 (0.1 to 0.6) | 1.2 (0.7 to 1.8) | 60 (48.3 to 74.2) |
| Bangladesh | Females | 0.3 (0.1 to 0.6) | 0.7 (0.4 to 1.2) | 47.2 (36 to 61.8) |
| Bangladesh | Both sexes | 0.3 (0.1 to 0.5) | 1 (0.6 to 1.4) | 53.7 (44.8 to 65.7) |
| Belarus | Males | 1.6 (0.8 to 3.1) | 0.4 (0.2 to 0.5) | 170.8 (96.1 to 289.4) |
| Belarus | Females | 1.3 (0.7 to 2.3) | 0.3 (0.2 to 0.4) | 127.2 (71.7 to 202.4) |
| Belarus | Both sexes | 1.4 (0.8 to 2.4) | 0.3 (0.3 to 0.4) | 149.3 (97.7 to 220.9) |
| Belize | Males | 1.6 (1.1 to 2.3) | 1 (0.8 to 1.3) | 131.4 (67.7 to 213.5) |
| Belize | Females | 0.8 (0.5 to 1.5) | 0.8 (0.7 to 1) | 100.9 (63.9 to 148.3) |
| Belize | Both sexes | 1.2 (0.9 to 1.9) | 0.9 (0.8 to 1.1) | 116 (75.1 to 170.7) |
| Benin | Males | 2.6 (1.4 to 4) | 3.5 (2.1 to 5.4) | 164.8 (80.3 to 266.6) |
| Benin | Females | 2 (1.2 to 3.2) | 2.4 (1.5 to 3.7) | 125.2 (77.1 to 194.7) |
| Benin | Both sexes | 2.3 (1.6 to 3.4) | 2.9 (2 to 4.2) | 145 (84.5 to 218.9) |
| Bhutan | Males | 3.1 (1.4 to 6.2) | 1.4 (0.8 to 2.3) | 84 (67.4 to 107) |
| Bhutan | Females | 2.2 (1 to 4.4) | 0.9 (0.5 to 1.6) | 68.6 (54.9 to 83.4) |
| Bhutan | Both sexes | 2.6 (1.4 to 4.8) | 1.2 (0.8 to 1.8) | 76.4 (64.3 to 89.3) |
| Bolivia (Plurinational State of) | Males | 5.5 (2.9 to 8.9) | 2.9 (1.7 to 4.4) | 118.5 (72.8 to 179.4) |
| Bolivia (Plurinational State of) | Females | 3.3 (1.9 to 5.5) | 3.2 (2 to 5) | 103.2 (64.8 to 153.7) |
| Bolivia (Plurinational State of) | Both sexes | 4.5 (2.6 to 6.9) | 3 (2 to 4.5) | 110.9 (76.2 to 154.3) |
| Bosnia and Herzegovina | Males | 0.4 (0.1 to 1.1) | 0.6 (0.3 to 1) | 129.9 (53.7 to 217.4) |
| Bosnia and Herzegovina | Females | 1.1 (0.6 to 2) | 0.4 (0.2 to 0.6) | 129.9 (64.1 to 207) |
| Bosnia and Herzegovina | Both sexes | 0.7 (0.4 to 1.4) | 0.5 (0.3 to 0.8) | 129.9 (69 to 191.1) |
| Botswana | Males | 3.1 (1.8 to 5.2) | 0.8 (0.4 to 1.3) | 97.7 (54.7 to 159) |
| Botswana | Females | 2.4 (1.4 to 3.9) | 1.5 (0.9 to 2.2) | 69.9 (42 to 106.3) |
| Botswana | Both sexes | 2.8 (1.9 to 4.2) | 1.1 (0.7 to 1.6) | 84.1 (53.9 to 123.7) |
| Brazil | Males | 0.1 (0 to 0.2) | 1.1 (1 to 1.3) | 96.9 (53.3 to 164.5) |
| Brazil | Females | 1 (0.5 to 1.9) | 0.9 (0.8 to 1.1) | 70.9 (41.7 to 108.4) |
| Brazil | Both sexes | 0.6 (0.3 to 1) | 1 (0.9 to 1.2) | 84.2 (56 to 134.2) |
| Burkina Faso | Males | 3.1 (1.3 to 5.6) | 1 (0.6 to 1.7) | 29.6 (21 to 39.2) |
| Burkina Faso | Females | 3.4 (1.7 to 5.9) | 0.9 (0.6 to 1.3) | 26.6 (19.4 to 35.4) |
| Burkina Faso | Both sexes | 3.2 (1.8 to 5.6) | 1 (0.7 to 1.4) | 28.1 (22.7 to 34.8) |
| Burundi | Males | 2.1 (1.1 to 4.4) | 2.2 (1 to 3.8) | 103.1 (49.9 to 171.2) |
| Burundi | Females | 1.5 (0.8 to 2.4) | 1.6 (0.9 to 2.5) | 62.9 (35.4 to 92.7) |
| Burundi | Both sexes | 1.8 (1.2 to 3.2) | 1.9 (1.1 to 2.9) | 83.4 (47.4 to 125.1) |
| C?te d'Ivoire | Males | 0.1 (0 to 0.3) | 1.2 (0.7 to 1.8) | 103.9 (83.9 to 121.6) |
| C?te d'Ivoire | Females | 0.6 (0.3 to 1) | 0.7 (0.5 to 1.1) | 69.4 (57.6 to 82.6) |
| C?te d'Ivoire | Both sexes | 0.4 (0.2 to 0.6) | 1 (0.7 to 1.3) | 86.9 (75.3 to 98) |
| Cabo Verde | Males | 1.7 (0.8 to 3.3) | 1.7 (0.9 to 3.3) | 238.5 (126.7 to 365.5) |
| Cabo Verde | Females | 0.9 (0.4 to 1.8) | 0.8 (0.5 to 1.4) | 144.3 (85.3 to 213.7) |
| Cabo Verde | Both sexes | 1.3 (0.7 to 2.5) | 1.3 (0.8 to 2.1) | 194.5 (117.2 to 274.1) |
| Cambodia | Males | 1.8 (1.1 to 2.6) | 1.7 (1 to 2.8) | 122.9 (77.9 to 178.7) |
| Cambodia | Females | 1.5 (0.9 to 2.5) | 1.3 (0.8 to 2.1) | 96.1 (57.2 to 144.2) |
| Cambodia | Both sexes | 1.6 (1.1 to 2.5) | 1.5 (1 to 2.3) | 109.6 (76.2 to 150.6) |
| Cameroon | Males | 2.8 (1.6 to 4.8) | 1.6 (0.9 to 2.6) | 124.4 (54.5 to 213.5) |
| Cameroon | Females | 2.2 (1.2 to 3.7) | 1.2 (0.8 to 1.8) | 125.6 (58.7 to 204.9) |
| Cameroon | Both sexes | 2.5 (1.6 to 3.9) | 1.4 (0.9 to 2) | 125 (60 to 188.9) |
| Central African Republic | Males | 3.2 (1.6 to 5.7) | 2.3 (1.4 to 3.5) | 140.2 (79.3 to 222.8) |
| Central African Republic | Females | 2.1 (1.1 to 3.9) | 1.4 (0.8 to 2.1) | 105.9 (62.6 to 169.5) |
| Central African Republic | Both sexes | 2.7 (1.5 to 4.3) | 1.8 (1.2 to 2.6) | 123.4 (82.2 to 183.8) |
| Chad | Males | 6 (3.1 to 11.7) | 1.5 (0.9 to 2.1) | 92.5 (55.2 to 148.4) |
| Chad | Females | 5.1 (2.4 to 9.9) | 1.2 (0.7 to 1.8) | 70.3 (44.1 to 111.1) |
| Chad | Both sexes | 5.6 (3 to 10.5) | 1.3 (0.9 to 1.8) | 81.4 (53 to 112.1) |
| China | Males | 0 (0 to 0.2) | 1.7 (1.1 to 2.4) | 92.7 (50.8 to 153.1) |
| China | Females | 0.3 (0.1 to 0.5) | 1.2 (0.8 to 1.7) | 100.4 (65 to 152.6) |
| China | Both sexes | 0.2 (0.1 to 0.3) | 1.5 (1 to 1.9) | 96.5 (65.6 to 139.2) |
| Colombia | Males | 3.6 (2.6 to 5.5) | 1.8 (1.5 to 2) | 60.4 (38.1 to 92.2) |
| Colombia | Females | 2.6 (1.7 to 4) | 1.3 (1.1 to 1.6) | 45.6 (28.9 to 70.2) |
| Colombia | Both sexes | 3.1 (2.2 to 4.6) | 1.6 (1.4 to 1.7) | 53.3 (37.6 to 76.4) |
| Comoros | Males | 0.1 (0 to 0.3) | 1.3 (0.7 to 2.1) | 109.9 (52.1 to 187.9) |
| Comoros | Females | 0.4 (0.2 to 0.8) | 1.1 (0.7 to 1.7) | 100.6 (55.4 to 149.8) |
| Comoros | Both sexes | 0.3 (0.1 to 0.5) | 1.2 (0.7 to 1.7) | 105.4 (63.3 to 147.5) |
| Congo | Males | 2.4 (1.4 to 4.1) | 1.8 (1.1 to 2.7) | 49.8 (27.4 to 81.9) |
| Congo | Females | 1.4 (0.7 to 2.7) | 1 (0.6 to 1.6) | 29.2 (17.3 to 44.5) |
| Congo | Both sexes | 1.9 (1.1 to 3.2) | 1.4 (1 to 2) | 39.8 (24.1 to 59.9) |
| Costa Rica | Males | 1.6 (0.8 to 2.8) | 1.4 (1.2 to 1.7) | 196.7 (82.3 to 357.4) |
| Costa Rica | Females | 1 (0.6 to 1.8) | 1.4 (1.1 to 1.8) | 215.5 (99.2 to 364.2) |
| Costa Rica | Both sexes | 1.3 (0.8 to 2) | 1.4 (1.2 to 1.6) | 205.8 (120.2 to 315.2) |
| Cuba | Males | 1.8 (0.8 to 3.5) | 1.2 (0.9 to 1.5) | 109.2 (57.2 to 179.3) |
| Cuba | Females | 1.1 (0.5 to 2.4) | 0.7 (0.5 to 0.9) | 88.8 (54.8 to 137.7) |
| Cuba | Both sexes | 1.5 (0.8 to 2.9) | 1 (0.8 to 1.1) | 99.3 (60.9 to 142.4) |
| Democratic People's Republic of Korea | Males | 2.1 (1.4 to 3.2) | 1.6 (0.7 to 2.7) | 143.7 (71 to 240.1) |
| Democratic People's Republic of Korea | Females | 1.6 (1 to 2.8) | 1.6 (0.8 to 2.5) | 106.5 (56.9 to 165.4) |
| Democratic People's Republic of Korea | Both sexes | 1.9 (1.2 to 3) | 1.6 (0.9 to 2.4) | 125.8 (74.8 to 197.5) |
| Democratic Republic of the Congo | Males | 3.9 (2 to 6.9) | 1.9 (1.1 to 3) | 5.6 (1.5 to 13.8) |
| Democratic Republic of the Congo | Females | 2.8 (1.2 to 5.4) | 1.1 (0.7 to 1.7) | 57 (29.5 to 101.2) |
| Democratic Republic of the Congo | Both sexes | 3.4 (1.6 to 6.1) | 1.5 (1 to 2.2) | 30.5 (15.7 to 53.6) |
| Djibouti | Males | 0.6 (0.1 to 1.6) | 1.4 (0.7 to 2.4) | 234.4 (140.3 to 363.4) |
| Djibouti | Females | 0.9 (0.5 to 1.6) | 1 (0.6 to 1.7) | 258.5 (163.6 to 401.9) |
| Djibouti | Both sexes | 0.8 (0.3 to 1.4) | 1.2 (0.7 to 1.9) | 246.2 (164.7 to 362.8) |
| Dominica | Males | 1.3 (0.6 to 2.7) | 1.9 (1.4 to 2.8) | 158.2 (92.7 to 246.2) |
| Dominica | Females | 0.7 (0.4 to 1.3) | 1.1 (0.7 to 1.5) | 93.4 (53.5 to 139.4) |
| Dominica | Both sexes | 1.1 (0.5 to 1.9) | 1.5 (1.1 to 2.1) | 126.3 (84.6 to 183.7) |
| Dominican Republic | Males | 1.8 (1.1 to 2.9) | 1 (0.6 to 1.6) | 62.9 (49 to 80) |
| Dominican Republic | Females | 1.4 (0.8 to 2.5) | 0.7 (0.4 to 1) | 53 (40.6 to 69.6) |
| Dominican Republic | Both sexes | 1.6 (1 to 2.6) | 0.8 (0.5 to 1.2) | 58 (47.7 to 69.7) |
| Ecuador | Males | 2 (1.2 to 3.3) | 3 (2.5 to 3.4) | 65.9 (46.3 to 91.7) |
| Ecuador | Females | 1.6 (0.9 to 3) | 2.5 (2.1 to 2.9) | 57.2 (40.4 to 77.3) |
| Ecuador | Both sexes | 1.8 (1.1 to 3.2) | 2.7 (2.5 to 3) | 61.7 (47.6 to 79.6) |
| Egypt | Males | 2.4 (1.3 to 3.8) | 1.3 (0.7 to 2.1) | 106.3 (58.5 to 169.7) |
| Egypt | Females | 1.8 (1 to 3.1) | 1.1 (0.6 to 1.7) | 89.5 (52.9 to 136.7) |
| Egypt | Both sexes | 2.1 (1.2 to 3.5) | 1.2 (0.8 to 1.7) | 98.2 (66.4 to 141) |
| El Salvador | Males | 1.6 (0.8 to 2.7) | 2.5 (1.4 to 3.5) | 84 (50.4 to 142.4) |
| El Salvador | Females | 1.3 (0.6 to 2.6) | 2.2 (1.4 to 3) | 71.7 (38 to 115.1) |
| El Salvador | Both sexes | 1.4 (0.7 to 2.7) | 2.4 (1.5 to 3) | 78.1 (49.8 to 114) |
| Equatorial Guinea | Males | 2.2 (1.5 to 3.2) | 1.6 (0.9 to 2.5) | 126.4 (44.1 to 219.6) |
| Equatorial Guinea | Females | 1.8 (1.1 to 2.9) | 0.7 (0.4 to 1.2) | 65.8 (39.5 to 101.1) |
| Equatorial Guinea | Both sexes | 2 (1.4 to 3) | 1.2 (0.8 to 1.9) | 96.8 (45.8 to 151.6) |
| Eritrea | Males | 11 (5.4 to 17.2) | 2.9 (1.6 to 4.6) | 85.9 (53.6 to 138.4) |
| Eritrea | Females | 9.2 (5 to 16.5) | 2.1 (1.3 to 3.2) | 72.4 (48.3 to 108.9) |
| Eritrea | Both sexes | 10.1 (6 to 16.8) | 2.5 (1.6 to 3.7) | 79.1 (54.1 to 116.3) |
| Eswatini | Males | 0.7 (0.5 to 1) | 1.3 (0.8 to 2) | 113.9 (66.3 to 192.1) |
| Eswatini | Females | 1.6 (1.1 to 2.6) | 1.2 (0.8 to 1.8) | 77 (41.3 to 129.1) |
| Eswatini | Both sexes | 1.1 (0.8 to 1.7) | 1.2 (0.8 to 1.7) | 95.7 (64.1 to 143.8) |
| Ethiopia | Males | 3.3 (1.9 to 5.5) | 5.9 (3.5 to 9.3) | 118.1 (57.6 to 208.1) |
| Ethiopia | Females | 2.3 (1.2 to 4.6) | 3 (1.8 to 4.8) | 88.1 (49.6 to 143) |
| Ethiopia | Both sexes | 2.8 (1.6 to 5) | 4.5 (3 to 6.6) | 103.4 (62.2 to 162.2) |
| Fiji | Males | 4.5 (3.5 to 5.9) | 0.4 (0 to 1) | 156.5 (87.7 to 266) |
| Fiji | Females | 3.8 (2.8 to 5.6) | 0.5 (0.3 to 0.8) | 116.3 (64.1 to 199.9) |
| Fiji | Both sexes | 4.2 (3.3 to 5.5) | 0.4 (0.2 to 0.8) | 136.8 (87.6 to 210.7) |
| Gabon | Males | 1.3 (0.9 to 1.8) | 1.9 (1.2 to 3) | 156.8 (66.9 to 265.1) |
| Gabon | Females | 1.5 (1 to 2.2) | 0.9 (0.6 to 1.5) | 195.1 (90.1 to 334.2) |
| Gabon | Both sexes | 1.4 (1 to 1.9) | 1.4 (1 to 2) | 175.9 (98.3 to 259.6) |
| Gambia | Males | 2.9 (2 to 4.4) | 0.3 (0.2 to 0.5) | 160.7 (93.3 to 251.9) |
| Gambia | Females | 2.2 (1.3 to 3.9) | 0.5 (0.3 to 0.8) | 158.7 (97.3 to 259.3) |
| Gambia | Both sexes | 2.5 (1.7 to 4) | 0.4 (0.3 to 0.6) | 159.8 (109.5 to 217.9) |
| Georgia | Males | 4.6 (2.8 to 7.1) | 0.5 (0.4 to 0.7) | 214.5 (140.4 to 314.3) |
| Georgia | Females | 3.8 (2.2 to 6.7) | 0.4 (0.3 to 0.6) | 118.2 (69.5 to 189.6) |
| Georgia | Both sexes | 4.2 (2.6 to 6.6) | 0.5 (0.4 to 0.6) | 166.1 (115.8 to 232.1) |
| Ghana | Males | 4.4 (3 to 6.7) | 1.7 (1 to 3) | 107.5 (64.5 to 174.3) |
| Ghana | Females | 3.9 (2.2 to 7) | 0.8 (0.5 to 1.3) | 71.4 (43.6 to 117.7) |
| Ghana | Both sexes | 4.1 (2.7 to 6.6) | 1.3 (0.8 to 1.9) | 90.2 (61.6 to 128.4) |
| Grenada | Males | 3 (1.4 to 5) | 0.4 (0.3 to 0.6) | 478.6 (280.3 to 752.3) |
| Grenada | Females | 4.8 (2.8 to 7.5) | 0.9 (0.7 to 1.2) | 241.6 (141.8 to 384) |
| Grenada | Both sexes | 3.9 (2.5 to 6) | 0.7 (0.6 to 0.8) | 362.5 (242.6 to 529.3) |
| Guatemala | Males | 5.8 (4.4 to 8) | 2.8 (2.2 to 3.5) | 137.2 (86.1 to 211.1) |
| Guatemala | Females | 5.3 (3.6 to 8) | 2.3 (1.8 to 2.8) | 71.1 (46.1 to 104.6) |
| Guatemala | Both sexes | 5.6 (4.1 to 8) | 2.5 (2.1 to 2.9) | 103.7 (73.5 to 144.7) |
| Guinea | Males | 1.7 (0.9 to 3.1) | 0.4 (0.2 to 0.7) | 179.8 (119.4 to 254.2) |
| Guinea | Females | 1.3 (0.7 to 2.2) | 0.9 (0.6 to 1.4) | 138.4 (92.8 to 200.8) |
| Guinea | Both sexes | 1.5 (0.9 to 2.3) | 0.7 (0.4 to 0.9) | 159.7 (111.6 to 213.3) |
| Guinea-Bissau | Males | 3 (1.8 to 4.4) | 1.4 (0.9 to 2.1) | 194.6 (97.3 to 321.7) |
| Guinea-Bissau | Females | 2.6 (1.7 to 4.3) | 1.3 (0.8 to 1.9) | 156.3 (85.5 to 240.3) |
| Guinea-Bissau | Both sexes | 2.8 (1.9 to 4) | 1.4 (0.9 to 1.9) | 175.6 (102.4 to 264.2) |
| Haiti | Males | 3.4 (1.6 to 5.9) | 2.1 (1 to 3.6) | 75.5 (57.2 to 97.5) |
| Haiti | Females | 2.7 (1.2 to 5.7) | 3.4 (2 to 5.1) | 54.1 (42.3 to 68.7) |
| Haiti | Both sexes | 3.1 (1.6 to 5.6) | 2.8 (1.8 to 4.2) | 65.3 (54.2 to 78.4) |
| Honduras | Males | 1.9 (1.4 to 2.6) | 1.1 (0.6 to 1.9) | 11.9 (3.6 to 30.6) |
| Honduras | Females | 1.7 (1.1 to 2.6) | 1.2 (0.8 to 1.9) | 42.4 (22.4 to 72.3) |
| Honduras | Both sexes | 1.8 (1.3 to 2.5) | 1.2 (0.8 to 1.7) | 26.3 (14.2 to 43.6) |
| India | Males | 4.7 (2.6 to 7.3) | 0.7 (0.5 to 1.1) | 137.8 (74.2 to 221.9) |
| India | Females | 4.5 (2.8 to 7.3) | 0.6 (0.3 to 0.8) | 95.3 (53.9 to 153.5) |
| India | Both sexes | 4.6 (3.1 to 7.3) | 0.7 (0.5 to 0.9) | 117.3 (75.6 to 172.8) |
| Indonesia | Males | 5.4 (4.2 to 7) | 2.1 (1.2 to 3.4) | 6.9 (2 to 17.8) |
| Indonesia | Females | 4.3 (3.1 to 6.7) | 1.5 (0.7 to 2.4) | 31.8 (17.6 to 53.8) |
| Indonesia | Both sexes | 4.9 (3.8 to 6.7) | 1.8 (1 to 2.6) | 18.9 (10.4 to 32.9) |
| Iran (Islamic Republic of) | Males | 1.3 (0.9 to 2) | 2 (1.2 to 3.1) | 56 (29.5 to 96.4) |
| Iran (Islamic Republic of) | Females | 0.9 (0.6 to 1.6) | 1.9 (1.2 to 3.1) | 27.7 (15.2 to 45.4) |
| Iran (Islamic Republic of) | Both sexes | 1.1 (0.8 to 1.8) | 2 (1.3 to 2.7) | 43.1 (24.4 to 67.4) |
| Iraq | Males | 4.5 (2.6 to 7.1) | 1.6 (0.9 to 2.6) | 291.5 (177.8 to 455.8) |
| Iraq | Females | 5 (3.2 to 8.1) | 1.3 (0.8 to 2.1) | 195.4 (120.4 to 305.7) |
| Iraq | Both sexes | 4.8 (3.1 to 7) | 1.5 (0.9 to 2.1) | 244 (168.1 to 352.9) |
| Jamaica | Males | 2.1 (1 to 3.5) | 1.2 (0.9 to 1.5) | 94.2 (72.3 to 120) |
| Jamaica | Females | 2 (1.1 to 3.4) | 0.8 (0.6 to 1) | 67.4 (50.7 to 85.5) |
| Jamaica | Both sexes | 2.1 (1.1 to 3.3) | 1 (0.8 to 1.2) | 81 (65.8 to 97) |
| Jordan | Males | 1.9 (0.7 to 4) | 0.6 (0.2 to 1) | 52.2 (18.5 to 106.5) |
| Jordan | Females | 2.8 (1.1 to 5.9) | 0.7 (0.3 to 1.1) | 81.5 (52 to 123.1) |
| Jordan | Both sexes | 2.3 (1 to 4.8) | 0.6 (0.3 to 0.9) | 67 (40.5 to 104.6) |
| Kazakhstan | Males | 3.8 (1.6 to 7) | 0.8 (0.6 to 1) | 2.3 (0.2 to 9.3) |
| Kazakhstan | Females | 4.2 (1.8 to 9) | 0.6 (0.5 to 0.8) | 13.5 (7.4 to 23.1) |
| Kazakhstan | Both sexes | 4 (1.9 to 7.2) | 0.7 (0.6 to 0.8) | 7.7 (3.8 to 14.1) |
| Kenya | Males | 3.9 (2.5 to 6.1) | 1.1 (0.7 to 1.7) | 223.2 (179.6 to 278.2) |
| Kenya | Females | 3.8 (2.1 to 7.1) | 0.8 (0.4 to 1.2) | 181 (144.9 to 223.7) |
| Kenya | Both sexes | 3.9 (2.3 to 6.6) | 0.9 (0.6 to 1.3) | 202.3 (172 to 233.2) |
| Kiribati | Males | 2.9 (2 to 4.4) | 0.1 (0 to 0.2) | 150.1 (106.1 to 218) |
| Kiribati | Females | 1.8 (1.1 to 2.6) | 0.7 (0.3 to 1.2) | 84.7 (58.1 to 118.4) |
| Kiribati | Both sexes | 2.3 (1.7 to 3.4) | 0.3 (0.2 to 0.6) | 118.5 (89.3 to 161.1) |
| Kyrgyzstan | Males | 2.1 (1.7 to 2.9) | 0.8 (0.6 to 1) | 71.8 (37.1 to 123.6) |
| Kyrgyzstan | Females | 2 (1.3 to 3.3) | 0.4 (0.3 to 0.5) | 48.3 (27.6 to 76.9) |
| Kyrgyzstan | Both sexes | 2.1 (1.6 to 3) | 0.6 (0.5 to 0.7) | 60.3 (37.5 to 89) |
| Lao People's Democratic Republic | Males | 2.9 (1.4 to 5.1) | 1.9 (1.1 to 3.3) | 144 (116.5 to 174.1) |
| Lao People's Democratic Republic | Females | 2.7 (1.4 to 4.6) | 1.4 (0.8 to 2.5) | 106.6 (84.7 to 128.5) |
| Lao People's Democratic Republic | Both sexes | 2.8 (1.6 to 4.4) | 1.7 (1.1 to 2.6) | 125.9 (108.5 to 144.4) |
| Lebanon | Males | 1.8 (0.9 to 2.8) | 1.8 (0.8 to 3) | 242.2 (133.2 to 386.6) |
| Lebanon | Females | 2 (1.2 to 3.2) | 1.3 (0.7 to 2) | 172.5 (110.8 to 263.2) |
| Lebanon | Both sexes | 1.9 (1.3 to 2.7) | 1.6 (0.9 to 2.3) | 208.5 (131.5 to 304) |
| Lesotho | Males | 1.2 (0.7 to 1.9) | 0.8 (0.5 to 1.4) | 190.8 (118.3 to 290.8) |
| Lesotho | Females | 0.9 (0.6 to 1.5) | 0.6 (0.3 to 0.9) | 112.8 (69.2 to 171.6) |
| Lesotho | Both sexes | 1.1 (0.7 to 1.5) | 0.7 (0.5 to 1) | 152.2 (103.3 to 215.3) |
| Liberia | Males | 6.5 (2.7 to 12.4) | 1.3 (0.7 to 2.2) | 397.4 (202.4 to 544) |
| Liberia | Females | 5.6 (2.3 to 12) | 1.1 (0.7 to 1.7) | 304.2 (173.6 to 400.3) |
| Liberia | Both sexes | 6.1 (2.7 to 11.6) | 1.2 (0.8 to 1.7) | 351.6 (221.7 to 453.8) |
| Libya | Males | 2.6 (1.2 to 5.3) | 1.5 (0.7 to 2.6) | 139.8 (72.2 to 209.3) |
| Libya | Females | 2.1 (1 to 4.2) | 1.5 (0.7 to 2.4) | 111.6 (67.1 to 166.9) |
| Libya | Both sexes | 2.4 (1.2 to 4.5) | 1.5 (0.7 to 2.3) | 126.2 (80.7 to 173.5) |
| Madagascar | Males | 4.2 (2.1 to 7.2) | 1.6 (0.8 to 2.6) | 65.7 (32.9 to 108) |
| Madagascar | Females | 4 (1.8 to 8.7) | 1.1 (0.7 to 1.6) | 120 (75 to 181) |
| Madagascar | Both sexes | 4.1 (2.2 to 7.8) | 1.3 (0.8 to 2) | 92.7 (60.4 to 133.5) |
| Malawi | Males | 3.5 (1.4 to 6.5) | 0.5 (0.2 to 0.8) | 26.9 (16.1 to 43) |
| Malawi | Females | 4 (1.8 to 6.7) | 0.6 (0.3 to 0.9) | 39.7 (24.7 to 62) |
| Malawi | Both sexes | 3.7 (2.1 to 5.8) | 0.5 (0.3 to 0.8) | 33.3 (22.4 to 48.2) |
| Malaysia | Males | 2.8 (1.6 to 4.3) | 0.8 (0.4 to 1.5) | 135.9 (76.7 to 271.7) |
| Malaysia | Females | 1.7 (0.9 to 2.6) | 0.6 (0.3 to 0.9) | 68.8 (43.3 to 115.7) |
| Malaysia | Both sexes | 2.2 (1.5 to 3.2) | 0.7 (0.4 to 1.1) | 103.1 (65.7 to 173) |
| Maldives | Males | 3.9 (2.5 to 5.6) | 0.7 (0.4 to 1.2) | 133.5 (71 to 212.1) |
| Maldives | Females | 2.1 (1.3 to 3.5) | 0.3 (0.2 to 0.5) | 110.2 (62.9 to 172.5) |
| Maldives | Both sexes | 3 (2.1 to 4.1) | 0.5 (0.3 to 0.8) | 122.1 (71.6 to 170.4) |
| Mali | Males | 6.3 (2.8 to 11.8) | 1.5 (0.5 to 2.5) | 133.4 (66.4 to 212.8) |
| Mali | Females | 5 (2.1 to 11.2) | 0.8 (0.5 to 1.2) | 87.7 (54.1 to 135) |
| Mali | Both sexes | 5.7 (2.7 to 10.4) | 1.1 (0.6 to 1.8) | 110.7 (66.8 to 163.6) |
| Marshall Islands | Males | 3 (1.4 to 5.1) | 0.1 (0 to 0.3) | 37.7 (21.4 to 64) |
| Marshall Islands | Females | 3.5 (1.6 to 6.2) | 0.4 (0.2 to 0.7) | 23.7 (13.5 to 36.5) |
| Marshall Islands | Both sexes | 3.3 (1.8 to 4.9) | 0.2 (0.1 to 0.5) | 31.1 (20.3 to 49.4) |
| Mauritania | Males | 1.6 (0.9 to 2.6) | 1.2 (0.7 to 1.9) | 81.2 (54.4 to 114.5) |
| Mauritania | Females | 1.4 (0.7 to 2.2) | 1 (0.7 to 1.6) | 39.5 (20.7 to 63.1) |
| Mauritania | Both sexes | 1.5 (0.9 to 2.2) | 1.1 (0.8 to 1.5) | 60.6 (39.9 to 80.2) |
| Mauritius | Males | 2.2 (1.3 to 3.9) | 0.7 (0.5 to 0.8) | 146.6 (94 to 220.4) |
| Mauritius | Females | 1.6 (0.9 to 3) | 0.6 (0.5 to 0.7) | 85.8 (50.3 to 132.6) |
| Mauritius | Both sexes | 1.9 (1.2 to 3.1) | 0.6 (0.5 to 0.7) | 115.9 (82.6 to 165.9) |
| Mexico | Males | 0.6 (0.3 to 1.1) | 3 (2.7 to 3.4) | 92 (81.9 to 104.4) |
| Mexico | Females | 0.6 (0.3 to 1) | 2.2 (1.9 to 2.5) | 77.5 (67.1 to 88.3) |
| Mexico | Both sexes | 0.6 (0.3 to 1) | 2.6 (2.4 to 2.8) | 84.9 (76.8 to 94.4) |
| Micronesia (Federated States of) | Males | 8.5 (5 to 13.9) | 0 (0 to 0.1) | 66 (37.5 to 109.8) |
| Micronesia (Federated States of) | Females | 4.2 (2.6 to 7) | 0.1 (0.1 to 0.3) | 45.4 (28.5 to 70.4) |
| Micronesia (Federated States of) | Both sexes | 6.4 (4.4 to 9.9) | 0.1 (0 to 0.2) | 55.7 (37.1 to 81.2) |
| Mongolia | Males | 3 (1.9 to 4.7) | 0.9 (0.4 to 1.5) | 157.4 (98.1 to 247) |
| Mongolia | Females | 1.5 (0.9 to 2.4) | 0.6 (0.3 to 0.9) | 77.3 (47.3 to 124.8) |
| Mongolia | Both sexes | 2.2 (1.5 to 3.2) | 0.7 (0.5 to 1.1) | 116.6 (82.6 to 163.1) |
| Montenegro | Males | 2 (1 to 3.3) | 0.9 (0.7 to 1.4) | 139.5 (87.6 to 197.7) |
| Montenegro | Females | 1.6 (1 to 2.5) | 0.8 (0.5 to 1.1) | 103.2 (66.5 to 136) |
| Montenegro | Both sexes | 1.8 (1.1 to 2.6) | 0.9 (0.7 to 1.1) | 122.5 (82.1 to 158.5) |
| Morocco | Males | 2.7 (1.7 to 4.1) | 0.3 (0.2 to 0.6) | 73.4 (41.5 to 117.6) |
| Morocco | Females | 1.6 (0.9 to 2.5) | 0.3 (0.2 to 0.5) | 60.8 (34.1 to 96.9) |
| Morocco | Both sexes | 2.1 (1.4 to 3.1) | 0.3 (0.2 to 0.5) | 67.3 (42.5 to 97.1) |
| Mozambique | Males | 7 (3.3 to 13.3) | 0.6 (0.2 to 1.3) | 141.3 (85.6 to 204.9) |
| Mozambique | Females | 8.4 (3.2 to 18.1) | 1 (0.6 to 1.4) | 118.5 (79.1 to 161.4) |
| Mozambique | Both sexes | 7.7 (3.4 to 14.7) | 0.8 (0.5 to 1.2) | 129.9 (88.3 to 169.9) |
| Myanmar | Males | 2.4 (1.2 to 4.1) | 2.1 (1.2 to 3.5) | 34.1 (24 to 44.8) |
| Myanmar | Females | 1.8 (1 to 2.8) | 1.6 (0.9 to 2.5) | 74.1 (57.5 to 96.1) |
| Myanmar | Both sexes | 2.1 (1.3 to 3.3) | 1.8 (1.2 to 2.7) | 53.4 (43.6 to 64.5) |
| Namibia | Males | 2.7 (1.2 to 5) | 1.2 (0.6 to 1.9) | 96.5 (74.5 to 123) |
| Namibia | Females | 2.1 (1 to 3.7) | 1.2 (0.7 to 1.9) | 59.3 (43.4 to 74.9) |
| Namibia | Both sexes | 2.4 (1.3 to 4.2) | 1.2 (0.8 to 1.7) | 78.6 (64 to 93) |
| Nepal | Males | 2.3 (1.2 to 3.7) | 1.1 (0.7 to 1.9) | 33.2 (3.9 to 81.7) |
| Nepal | Females | 1.5 (1 to 2.4) | 0.8 (0.4 to 1.2) | 42.9 (23.6 to 74.4) |
| Nepal | Both sexes | 1.9 (1.2 to 3) | 0.9 (0.6 to 1.5) | 37.9 (16.5 to 68.8) |
| Nicaragua | Males | 3.1 (1.4 to 5.5) | 2.2 (1.5 to 3.1) | 153.2 (67.6 to 253.8) |
| Nicaragua | Females | 2.3 (1.4 to 3.6) | 1.7 (1.2 to 2.5) | 165.9 (74.9 to 273.4) |
| Nicaragua | Both sexes | 2.7 (1.5 to 4.2) | 2 (1.4 to 2.6) | 159.4 (89.8 to 233.2) |
| Niger | Males | 2 (1.2 to 3.2) | 2.3 (1.2 to 3.8) | 142.8 (122.1 to 162.2) |
| Niger | Females | 1.3 (0.8 to 2.3) | 1.9 (1 to 2.9) | 109.3 (92.3 to 133.1) |
| Niger | Both sexes | 1.7 (1 to 2.7) | 2.1 (1.2 to 3.2) | 126.4 (113.8 to 138.7) |
| Nigeria | Males | 0.9 (0.3 to 1.8) | 1.6 (1 to 2.5) | 137.8 (77.5 to 244.2) |
| Nigeria | Females | 1.5 (0.9 to 2.3) | 0.9 (0.6 to 1.3) | 65.8 (42 to 103.8) |
| Nigeria | Both sexes | 1.2 (0.7 to 1.8) | 1.2 (0.9 to 1.7) | 102.3 (64.3 to 155.9) |
| North Macedonia | Males | 1.7 (1 to 2.8) | 0.8 (0.6 to 1.1) | 1.3 (0 to 5.4) |
| North Macedonia | Females | 1.2 (0.7 to 1.9) | 0.7 (0.5 to 1) | 1.8 (0 to 7.2) |
| North Macedonia | Both sexes | 1.4 (1 to 2.3) | 0.8 (0.6 to 1) | 1.6 (0.1 to 5.8) |
| Pakistan | Males | 2.5 (1.4 to 4) | 1 (0.6 to 1.7) | 40 (29.8 to 52.9) |
| Pakistan | Females | 1.2 (0.7 to 2.1) | 0.9 (0.5 to 1.4) | 36 (27.5 to 47.6) |
| Pakistan | Both sexes | 1.9 (1.1 to 3) | 0.9 (0.6 to 1.4) | 38.1 (30.9 to 46) |
| Palestine | Males | 4.2 (2.4 to 6.8) | 0.9 (0.5 to 1.6) | 95.9 (51.8 to 152.7) |
| Palestine | Females | 3 (2 to 4.8) | 0.7 (0.4 to 1) | 100.3 (59.1 to 154.8) |
| Palestine | Both sexes | 3.6 (2.4 to 5.3) | 0.8 (0.6 to 1.2) | 98.1 (62.2 to 141.5) |
| Papua New Guinea | Males | 3.2 (2 to 4.9) | 0.2 (0.1 to 0.7) | 54.6 (42.3 to 66.9) |
| Papua New Guinea | Females | 1.9 (1.2 to 3) | 0.7 (0.3 to 1.2) | 46.7 (36.7 to 58.9) |
| Papua New Guinea | Both sexes | 2.6 (1.8 to 3.7) | 0.4 (0.2 to 0.8) | 50.6 (42.7 to 58.2) |
| Paraguay | Males | 1.2 (0.7 to 2.1) | 1.8 (1.1 to 2.6) | 89.8 (55.3 to 156.2) |
| Paraguay | Females | 2.3 (1.4 to 3.4) | 1.5 (1 to 2) | 63 (36.3 to 99.8) |
| Paraguay | Both sexes | 1.8 (1.1 to 2.5) | 1.6 (1.1 to 2.1) | 76.8 (51.2 to 119.6) |
| Peru | Males | 1.2 (0.7 to 2) | 4.9 (2.5 to 6.7) | 120 (72.8 to 188.5) |
| Peru | Females | 1 (0.6 to 1.5) | 3.7 (2.1 to 4.9) | 90.4 (57.9 to 132.8) |
| Peru | Both sexes | 1.1 (0.7 to 1.7) | 4.3 (2.7 to 5.6) | 106 (74.6 to 141.7) |
| Philippines | Males | 2.2 (1.1 to 4) | 1.7 (0.9 to 2.6) | 46.1 (18 to 83.2) |
| Philippines | Females | 1.7 (0.9 to 2.8) | 1.4 (0.8 to 2) | 56.5 (29.2 to 93) |
| Philippines | Both sexes | 2 (1.1 to 3) | 1.5 (1 to 2.1) | 51.2 (26.4 to 78.7) |
| Republic of Moldova | Males | 2.1 (1.3 to 3.1) | 0.7 (0.5 to 0.8) | 168.8 (84.8 to 284.8) |
| Republic of Moldova | Females | 1.6 (1 to 2.4) | 0.6 (0.4 to 0.7) | 137.2 (82.1 to 207.4) |
| Republic of Moldova | Both sexes | 1.9 (1.3 to 2.6) | 0.6 (0.5 to 0.7) | 153.7 (91.5 to 226.6) |
| Rwanda | Males | 1.7 (1 to 2.7) | 2 (1 to 3.2) | 5.9 (1.6 to 15.6) |
| Rwanda | Females | 1.1 (0.7 to 1.8) | 1.5 (0.9 to 2.4) | 23 (11.9 to 39.1) |
| Rwanda | Both sexes | 1.4 (0.9 to 2) | 1.8 (1 to 2.6) | 14.2 (7.2 to 26.3) |
| Saint Lucia | Males | 2.1 (1.1 to 3.5) | 0.7 (0.5 to 1) | 129.5 (72.4 to 215.1) |
| Saint Lucia | Females | 1.6 (0.9 to 2.5) | 0.4 (0.3 to 0.6) | 99.7 (65.2 to 142.5) |
| Saint Lucia | Both sexes | 1.9 (1.1 to 2.8) | 0.6 (0.5 to 0.7) | 114.8 (75.9 to 163.7) |
| Saint Vincent and the Grenadines | Males | 1.9 (1.1 to 3) | 0.8 (0.6 to 1.1) | 239.8 (214.7 to 271.1) |
| Saint Vincent and the Grenadines | Females | 1.6 (1 to 2.4) | 0.9 (0.7 to 1.1) | 175.9 (154 to 199.9) |
| Saint Vincent and the Grenadines | Both sexes | 1.7 (1.1 to 2.5) | 0.8 (0.7 to 1) | 208.2 (190.1 to 226.8) |
| Samoa | Males | 1.6 (0.9 to 2.5) | 0 (0 to 0.1) | 121 (90.4 to 157.6) |
| Samoa | Females | 1.2 (0.8 to 2) | 0 (0 to 0.1) | 74.8 (56.3 to 100.1) |
| Samoa | Both sexes | 1.4 (0.9 to 2) | 0 (0 to 0.1) | 98.9 (81.3 to 124.1) |
| Sao Tome and Principe | Males | 1.5 (0.9 to 2.5) | 0.3 (0.1 to 0.4) | 63 (45.4 to 85.1) |
| Sao Tome and Principe | Females | 1.3 (0.8 to 1.9) | 0.6 (0.4 to 1) | 68.7 (52.8 to 86.7) |
| Sao Tome and Principe | Both sexes | 1.4 (1 to 2) | 0.4 (0.3 to 0.6) | 65.8 (54.5 to 80.5) |
| Senegal | Males | 1.4 (0.9 to 2) | 1.4 (0.9 to 2.3) | 171.4 (98.8 to 276.1) |
| Senegal | Females | 1.2 (0.8 to 2) | 1.1 (0.7 to 1.6) | 118.7 (57.5 to 193.6) |
| Senegal | Both sexes | 1.3 (0.9 to 2) | 1.3 (0.9 to 1.7) | 145.8 (85 to 213.2) |
| Serbia | Males | 0.5 (0.3 to 0.8) | 0.5 (0.3 to 0.8) | 199.1 (114.4 to 283.7) |
| Serbia | Females | 0.7 (0.4 to 1.1) | 0.3 (0.2 to 0.5) | 175.8 (118 to 238.8) |
| Serbia | Both sexes | 0.6 (0.4 to 0.9) | 0.4 (0.2 to 0.6) | 187.7 (124.2 to 239.5) |
| Sierra Leone | Males | 1.8 (1 to 2.8) | 1.6 (0.8 to 2.5) | 97.3 (59.8 to 148.2) |
| Sierra Leone | Females | 1.9 (1.1 to 3) | 1.2 (0.8 to 1.8) | 60.1 (39.6 to 85.8) |
| Sierra Leone | Both sexes | 1.8 (1.2 to 2.6) | 1.4 (0.9 to 2) | 79.4 (56.9 to 109.8) |
| Solomon Islands | Males | 2 (1.2 to 3.1) | 0.1 (0 to 0.2) | 146.3 (63.8 to 248.3) |
| Solomon Islands | Females | 1.7 (1.1 to 2.5) | 0.4 (0.2 to 0.6) | 106.7 (58.9 to 167.6) |
| Solomon Islands | Both sexes | 1.9 (1.3 to 2.6) | 0.2 (0.1 to 0.4) | 127.4 (74.2 to 185.1) |
| Somalia | Males | 1.4 (0.8 to 2.3) | 1.7 (0.8 to 2.9) | 77.3 (43.8 to 129.8) |
| Somalia | Females | 0.4 (0.3 to 0.7) | 1.3 (0.7 to 2) | 56.7 (34.5 to 84.1) |
| Somalia | Both sexes | 0.9 (0.6 to 1.4) | 1.5 (0.9 to 2.4) | 67.2 (45.5 to 99) |
| South Africa | Males | 3.1 (1.6 to 6.8) | 0.8 (0.4 to 1.2) | 180.7 (82.9 to 317.2) |
| South Africa | Females | 1.6 (0.8 to 2.8) | 0.6 (0.4 to 0.9) | 134.1 (78.5 to 202.7) |
| South Africa | Both sexes | 2.4 (1.3 to 4.6) | 0.7 (0.5 to 0.9) | 157.3 (87.3 to 236.1) |
| South Sudan | Males | 5.4 (3.3 to 8.4) | 2 (1 to 3.4) | 103.6 (61.4 to 161.7) |
| South Sudan | Females | 3.6 (2.1 to 5.7) | 1.7 (1 to 2.5) | 96.6 (62.6 to 145.3) |
| South Sudan | Both sexes | 4.5 (3 to 6.7) | 1.9 (1.1 to 2.7) | 100.2 (67 to 139.4) |
| Sri Lanka | Males | 2.4 (1.3 to 3.9) | 1 (0.7 to 1.4) | 37.7 (20.6 to 60.4) |
| Sri Lanka | Females | 1.8 (1.1 to 2.7) | 0.5 (0.2 to 0.8) | 73.4 (46.7 to 109.3) |
| Sri Lanka | Both sexes | 2.1 (1.3 to 3) | 0.8 (0.5 to 1) | 55.4 (36.3 to 77.1) |
| Sudan | Males | 3 (1.5 to 4.9) | 1.9 (0.8 to 3.2) | 240.2 (202.8 to 277.1) |
| Sudan | Females | 2.3 (1.4 to 3.6) | 2.4 (1.1 to 4.1) | 202.9 (172.4 to 235.5) |
| Sudan | Both sexes | 2.6 (1.5 to 3.9) | 2.2 (1.2 to 3.2) | 221.9 (200.9 to 247.2) |
| Suriname | Males | 2.3 (0.8 to 4) | 1.4 (0.7 to 2.3) | 80.3 (46 to 132.1) |
| Suriname | Females | 1.2 (0.7 to 1.8) | 1.2 (0.7 to 1.8) | 58.7 (35.4 to 87) |
| Suriname | Both sexes | 1.8 (0.8 to 2.8) | 1.3 (0.8 to 1.8) | 69.5 (43.4 to 99.9) |
| Syrian Arab Republic | Males | 2.6 (1.6 to 4) | 1.2 (0.5 to 2) | 127.2 (72.7 to 206.5) |
| Syrian Arab Republic | Females | 1.3 (0.8 to 2) | 0.8 (0.4 to 1.2) | 60.5 (32.6 to 102.9) |
| Syrian Arab Republic | Both sexes | 2 (1.4 to 2.7) | 1 (0.6 to 1.5) | 97.9 (60.8 to 158.4) |
| Tajikistan | Males | 0.7 (0.3 to 1.2) | 1.2 (0.6 to 2) | 27.3 (12.9 to 52) |
| Tajikistan | Females | 0.8 (0.5 to 1.4) | 0.9 (0.5 to 1.3) | 26.1 (14.4 to 41.6) |
| Tajikistan | Both sexes | 0.8 (0.4 to 1.2) | 1 (0.7 to 1.6) | 26.8 (16 to 41.2) |
| Thailand | Males | 1.8 (1 to 2.7) | 1.3 (0.6 to 2.1) | 190.2 (105.2 to 309.7) |
| Thailand | Females | 1.7 (1.1 to 2.6) | 0.8 (0.4 to 1.1) | 149.9 (86.3 to 240.7) |
| Thailand | Both sexes | 1.7 (1.2 to 2.4) | 1 (0.6 to 1.6) | 170.5 (112.5 to 240.8) |
| Timor-Leste | Males | 0.7 (0.4 to 1.1) | 2.3 (1.3 to 3.8) | 91 (56.9 to 140) |
| Timor-Leste | Females | 1.2 (0.8 to 1.9) | 1.8 (1 to 2.9) | 59.9 (35 to 96) |
| Timor-Leste | Both sexes | 0.9 (0.6 to 1.4) | 2.1 (1.4 to 2.9) | 75.7 (52.2 to 108.7) |
| Togo | Males | 1.1 (0.6 to 1.8) | 1.2 (0.6 to 1.9) | 80.3 (42.5 to 130.6) |
| Togo | Females | 0.8 (0.5 to 1.2) | 1.1 (0.7 to 1.6) | 24.1 (14.6 to 36.8) |
| Togo | Both sexes | 0.9 (0.6 to 1.4) | 1.1 (0.8 to 1.6) | 52.1 (30.6 to 77.8) |
| Tonga | Males | 1.4 (0.7 to 2.2) | 0.1 (0 to 0.3) | 9.4 (3 to 23.3) |
| Tonga | Females | 0.7 (0.5 to 1.2) | 0.2 (0.1 to 0.3) | 33.8 (16.5 to 59.7) |
| Tonga | Both sexes | 1 (0.6 to 1.5) | 0.1 (0.1 to 0.3) | 21.1 (11.2 to 37.5) |
| Tunisia | Males | 2.2 (1.3 to 3.4) | 1.6 (0.9 to 2.6) | 100.3 (53.1 to 155) |
| Tunisia | Females | 1.6 (1 to 2.4) | 1.1 (0.6 to 1.8) | 86.2 (53.4 to 129.9) |
| Tunisia | Both sexes | 1.9 (1.3 to 2.6) | 1.4 (0.9 to 2) | 93.4 (62.9 to 128.8) |
| Turkmenistan | Males | 3.5 (1.8 to 5.7) | 1.5 (1.1 to 1.9) | 62.9 (47.1 to 80.8) |
| Turkmenistan | Females | 2.7 (1.5 to 4.2) | 0.9 (0.7 to 1.2) | 33.2 (25.1 to 43.3) |
| Turkmenistan | Both sexes | 3.1 (1.8 to 4.7) | 1.2 (1 to 1.5) | 48.5 (39.1 to 59.8) |
| Tuvalu | Males | 2.4 (1.3 to 4) | 0.1 (0 to 0.4) | 9.9 (2.4 to 24.3) |
| Tuvalu | Females | 1.8 (1.1 to 2.6) | 0.5 (0.3 to 0.8) | 13.3 (6.4 to 24.3) |
| Tuvalu | Both sexes | 2.1 (1.4 to 3) | 0.3 (0.2 to 0.5) | 11.5 (5.2 to 21.3) |
| Uganda | Males | 2.5 (1.4 to 4.6) | 1.4 (0.7 to 2.5) | 96.3 (55.3 to 148.8) |
| Uganda | Females | 1.2 (0.7 to 1.8) | 1.1 (0.6 to 1.7) | 59.6 (35.1 to 97.1) |
| Uganda | Both sexes | 1.9 (1.2 to 2.9) | 1.2 (0.7 to 2) | 78.3 (51.5 to 114.7) |
| Ukraine | Males | 2 (1.1 to 3.3) | 0.7 (0.6 to 0.9) | 63.3 (35.5 to 94.9) |
| Ukraine | Females | 1.5 (0.9 to 2.4) | 0.6 (0.4 to 0.8) | 51 (32.7 to 76.4) |
| Ukraine | Both sexes | 1.8 (1.1 to 2.6) | 0.7 (0.6 to 0.8) | 57.2 (38.5 to 78) |
| United Republic of Tanzania | Males | 2.9 (1.5 to 4.9) | 1 (0.5 to 1.6) | 116.8 (58 to 198.3) |
| United Republic of Tanzania | Females | 2.3 (1.4 to 3.6) | 0.3 (0.2 to 0.4) | 85 (49.4 to 138.7) |
| United Republic of Tanzania | Both sexes | 2.6 (1.6 to 3.9) | 0.6 (0.4 to 0.9) | 102.2 (60.2 to 153.4) |
| Uzbekistan | Males | 1.8 (0.9 to 2.9) | 1.7 (1.4 to 2.1) | 75.1 (37.7 to 122.1) |
| Uzbekistan | Females | 1.5 (1 to 2.3) | 1.3 (1 to 1.5) | 40.3 (25.4 to 64) |
| Uzbekistan | Both sexes | 1.7 (1.1 to 2.3) | 1.5 (1.3 to 1.7) | 58 (34.3 to 84.1) |
| Vanuatu | Males | 0.4 (0.2 to 0.7) | 0.1 (0 to 0.2) | 62.7 (34.4 to 118.3) |
| Vanuatu | Females | 1 (0.6 to 1.5) | 0.3 (0.1 to 0.5) | 46.8 (26.5 to 75.3) |
| Vanuatu | Both sexes | 0.7 (0.5 to 1) | 0.2 (0.1 to 0.3) | 55.1 (34.9 to 89.2) |
| Viet Nam | Males | 0.2 (0.1 to 0.6) | 1.3 (0.8 to 2.1) | 39.3 (18.2 to 66.7) |
| Viet Nam | Females | 0.9 (0.5 to 1.4) | 0.9 (0.5 to 1.5) | 48.4 (28 to 77.1) |
| Viet Nam | Both sexes | 0.5 (0.3 to 0.8) | 1.1 (0.8 to 1.6) | 43.9 (26.2 to 68.1) |
| Yemen | Males | 3.5 (1.4 to 6.3) | 1.9 (0.8 to 3.1) | 110.8 (60.1 to 185.2) |
| Yemen | Females | 4.4 (1.9 to 7.8) | 2 (0.9 to 3.3) | 86.7 (54.8 to 134.4) |
| Yemen | Both sexes | 3.9 (2.1 to 6.3) | 2 (1.1 to 2.9) | 98.7 (63.7 to 141.3) |
| Zambia | Males | 3.6 (1.9 to 6.9) | 0.9 (0.4 to 1.5) | 97.3 (55.5 to 158.5) |
| Zambia | Females | 2.9 (1.2 to 5.9) | 0.5 (0.3 to 0.8) | 82.5 (53.7 to 125.1) |
| Zambia | Both sexes | 3.2 (1.7 to 6) | 0.7 (0.4 to 1) | 90 (61.4 to 124) |
| Zimbabwe | Males | 1.8 (1 to 2.8) | 1.1 (0.7 to 1.8) | 92.8 (43.2 to 158) |
| Zimbabwe | Females | 1.1 (0.7 to 1.5) | 0.8 (0.5 to 1.4) | 63.4 (34.5 to 95.6) |
| Zimbabwe | Both sexes | 1.4 (1 to 2) | 1 (0.7 to 1.4) | 78.3 (44.7 to 119.3) |
