# Supplementary material for: Burden of acute lymphoblastic leukemia in children and adolescents in low- and middle-income countries from 1990 to 2023 and projections to 2050: A systematic analysis from the global burden of disease study 2023
Source: PLoS One. 2026 Jun 2;21(6):e0350223. doi: 10.1371/journal.pone.0350223 (PMC13229300; doi:10.1371/journal.pone.0350223)
Supplement: S3 Table — (DOCX) [file pone.0350223.s003.docx]

# S3 Table. Average annual percent change of age-standardized incidence, mortality and DALYs rate from 1990 to 2023

| **Location_name** | **Sex** | **ASIR AAPC and 95%CI** | **ASMR AAPC and 95%CI** | **ASDR AAPC and 95%CI** |
| --- | --- | --- | --- | --- |
| World Bank Low Income | Both | -1.12 (-1.19 to -1.05) | -1.34 (-1.40 to -1.27) | -1.37 (-1.43 to -1.29) |
| World Bank Low Income | Male | -1.02 (-1.08 to -0.95) | -1.23 (-1.29 to -1.16) | -1.26 (-1.32 to -1.19) |
| World Bank Low Income | Female | -1.29 (-1.34 to -1.23) | -1.50 (-1.55 to -1.44) | -1.53 (-1.58 to -1.47) |
| World Bank Lower Middle Income | Both | -1.26 (-1.33 to -1.20) | -1.73 (-1.81 to -1.67) | -1.75 (-1.83 to -1.70) |
| World Bank Lower Middle Income | Male | -1.06 (-1.13 to -1.01) | -1.51 (-1.57 to -1.46) | -1.54 (-1.62 to -1.49) |
| World Bank Lower Middle Income | Female | -1.49 (-1.61 to -1.41) | -1.94 (-2.03 to -1.85) | -2.04 (-2.12 to -1.97) |
| World Bank Upper Middle Income | Both | -0.79 (-0.83 to -0.75) | -2.58 (-2.61 to -2.53) | -2.60 (-2.64 to -2.56) |
| World Bank Upper Middle Income | Male | -0.69 (-0.73 to -0.64) | -2.36 (-2.41 to -2.31) | -2.40 (-2.45 to -2.35) |
| World Bank Upper Middle Income | Female | -0.94 (-0.98 to -0.91) | -2.90 (-2.94 to -2.87) | -2.90 (-2.95 to -2.87) |

ASIR = age-standardised incidence rate, ASMR = age-standardised mortality rate, DALYs = disability-adjusted life years, ASDR = age-standardised rate of DALYs, UI = uncertainty intervals, AAPC = average annual percent changes
