# Supplementary material for: Burden of acute lymphoblastic leukemia in children and adolescents in low- and middle-income countries from 1990 to 2023 and projections to 2050: A systematic analysis from the global burden of disease study 2023
Source: PLoS One. 2026 Jun 2;21(6):e0350223. doi: 10.1371/journal.pone.0350223 (PMC13229300; doi:10.1371/journal.pone.0350223)
Supplement: S4 Table — (DOCX) [file pone.0350223.s004.docx]

# S4 Table. Age-standardized DALYs rates for acute lymphoblastic leukemia attributable to risk factors in 2023.

| **Location_name** | **Sex** | **Occupational exposure to benzene** | **Occupational exposure to formaldehyde** |
| --- | --- | --- | --- |
| World Bank Upper Middle Income | Male | 0.15(0.04 to 0.28) | 0.07 (0.03 to 0.12) |
| World Bank Upper Middle Income | Female | 0.14 (0.04 to 0.25) | 0.05 (0.02 to 0.09) |
| World Bank Upper Middle Income | Both | 0.15 (0.04 to 0.26) | 0.06 (0.03 to 0.09) |
| World Bank Lower Middle Income | Male | 0.06 (0.02 to 0.12) | 0.02 (0.01 to 0.04) |
| World Bank Lower Middle Income | Female | 0.05 (0.01 to 0.09) | 0.01 (0 to 0.03) |
| World Bank Lower Middle Income | Both | 0.05 (0.02 to 0.1) | 0.02 (0.01 to 0.03) |
| World Bank Low Income | Male | 0.13 (0.04 to 0.25) | 0.04 (0.01 to 0.09) |
| World Bank Low Income | Female | 0.12 (0.04 to 0.23) | 0.04 (0.01 to 0.07) |
| World Bank Low Income | Both | 0.12 (0.04 to 0.23) | 0.04 (0.02 to 0.07) |
