# Supplementary material for: Burden of acute lymphoblastic leukemia in children and adolescents in low- and middle-income countries from 1990 to 2023 and projections to 2050: A systematic analysis from the global burden of disease study 2023
Source: PLoS One. 2026 Jun 2;21(6):e0350223. doi: 10.1371/journal.pone.0350223 (PMC13229300; doi:10.1371/journal.pone.0350223)
Supplement: S5 Table — (DOCX) [file pone.0350223.s005.docx]

# S5 Table. Age-standardized DALYs rate of average annual percent change attributable to risk factors for acute lymphoblastic leukemia from 1990 to 2023

| **Location_name** | **Risk factor** | **AAPC, 95% CI** |
| --- | --- | --- |
| World Bank Low Income | Occupational exposure to benzene | -0.3 (-6 to 5.7) |
| World Bank Low Income | Occupational exposure to formaldehyde | -0.5 (-10 to 10) |
| World Bank Lower Middle Income | Occupational exposure to benzene | 0 (-8.2 to 9) |
| World Bank Lower Middle Income | Occupational exposure to formaldehyde | -0.3 (-13.9 to 15.4) |
| World Bank Upper Middle Income | Occupational exposure to benzene | -1.3 (-5.9 to 3.5) |
| World Bank Upper Middle Income | Occupational exposure to formaldehyde | -1.7 (-8.5 to 5.6) |

DALYs = disability-adjusted life years, AAPC = average annual percent change
