# Supplementary material for: Burden of acute lymphoblastic leukemia in children and adolescents in low- and middle-income countries from 1990 to 2023 and projections to 2050: A systematic analysis from the global burden of disease study 2023
Source: PLoS One. 2026 Jun 2;21(6):e0350223. doi: 10.1371/journal.pone.0350223 (PMC13229300; doi:10.1371/journal.pone.0350223)
Supplement: Attachment — Submitted filename: Review_ALL.pdf [file pone.0350223.s007.pdf]

1. The rationale for focusing exclusively on occupational benzene and formaldehyde exposure as risk factors in a pediatric and adolescent population requires clearer justification. Occupational exposure is biologically and contextually indirect for most individuals <20 years, and the manuscript does not explain how GBD attributes these risks to children and adolescents rather than parental or environmental exposure pathways. Clarification is needed on exposure assignment assumptions and their validity for this age group.
2. The description of the risk attribution framework is incomplete and internally inconsistent. The text states that “three types of risk factors” were identified, but only two (benzene and formaldehyde) are analyzed and reported. This discrepancy should be resolved, and the selection criteria for included risk factors should be explicitly stated in alignment with the GBD 2023 comparative risk assessment methodology.
3. The Bayesian Age–Period–Cohort (BAPC) model used for projections lacks sufficient methodological detail to allow reproducibility and critical appraisal. Key elements such as prior specifications, convergence diagnostics, uncertainty propagation, and model validation are not described (Methods, lines ~255–290). Given that future projections are a central conclusion; additional methodological transparency is required.
4. Several interpretive statements in the Discussion extend beyond what can be supported by GBD-level ecological data, particularly causal inferences linking socioeconomic development, diagnostic capacity, and incidence trends (Discussion, lines ~1020–1100). These interpretations should be more explicitly framed as hypotheses or contextual explanations rather than implied causal conclusions.
5. There are internal inconsistencies in terminology and disease naming (e.g., “acute lymphocytic leukemia” vs. “acute lymphoblastic leukemia”) that persist across the Abstract, Methods, and Results. Standardizing terminology throughout is necessary for clarity and editorial consistency.
6. The manuscript reports numerous precise numerical estimates and percentage changes without consistently linking them to figures or supplementary tables, making verification difficult. Key results, particularly trend estimates (AAPCs) and attributable DALY percentages, should be systematically cross-referenced to tables or figures as per PLOS ONE reporting standards.
7. The Discussion section is overly long and partially repetitive, with several paragraphs reiterating general background or speculative mechanisms already well described in the literature. Condensing this section and sharpening its focus on findings directly derived from the present analysis would improve clarity and editorial balance.
8. Language quality issues, including frequent typographical errors, non-standard phrasing, and repeated misspellings (e.g., “treatmalest,” “improvemalest,” “developmalest”), are present throughout the manuscript. While not a stylistic critique, the density of these errors materially affects readability and should be addressed through careful language editing prior to acceptance.
