# Supplementary material for: Burden of acute lymphoblastic leukemia in children and adolescents in low- and middle-income countries from 1990 to 2023 and projections to 2050: A systematic analysis from the global burden of disease study 2023
Source: PLoS One. 2026 Jun 2;21(6):e0350223. doi: 10.1371/journal.pone.0350223 (PMC13229300; doi:10.1371/journal.pone.0350223)
Supplement: Attachment — Submitted filename: Response to Reviewers#2docx..docx [file pone.0350223.s010.docx]

1. The rationale for focusing exclusively on occupational benzene and formaldehyde exposure as risk factors in a pediatric and adolescent population requires clearer justification. Occupational exposure is biologically and contextually indirect for most individuals <20 years, and the manuscript does not explain how GBD attributes	these	risks	to	children	and	adolescents	rather	than	parental	or environmental exposure pathways. Clarification is needed on exposure assignment assumptions and their validity for this age group.

Response:

We thank the reviewer for raising this critical methodological point. We agree that the link between occupational exposure and childhood/adolescent ALL is indirect and requires explicit justification, as direct occupational exposure is negligible for individuals under 20 years old in most settings.

Clarification of GBD Attribution Logic:

In the Global Burden of Disease (GBD) framework, when specific environmental monitoring data for children (e.g., ambient air concentrations of benzene/formaldehyde attributable to industrial sources) are unavailable or inconsistent globally, population-level occupational exposure estimates are often utilized as a proxy indicator for the overall intensity of industrial chemical release and environmental contamination in a given region.

The underlying assumption is not that children are working in these jobs, but that:

Environmental Spillover: High levels of occupational use in a region correlate with higher ambient environmental releases (via industrial emissions, waste, and transport).

Take-Home Exposure: Occupational exposures can be transferred to household environments via parents’ clothing and skin (the "take-home" pathway), indirectly exposing children.

Data Surrogacy: In the absence of granular environmental data for every country-year, occupational prevalence serves as the most robust available macro-level marker for the presence of these leukemogens in the human ecosystem.

Action Taken:

To address this concern and prevent misinterpretation, we have made the following substantial revisions:

Explicitly Stated the Proxy Nature: In the Methods section (under Exposure Assessment), we now clearly state that for the <20 age group, occupational exposure metrics serve as a surrogate for regional environmental burden and potential take-home exposure, rather than representing direct personal occupational contact.

Added Text: "For children and adolescents (<20 years), direct occupational exposure is minimal. Therefore, in this analysis, population-level occupational exposure estimates function as a proxy for the broader environmental availability of benzene and formaldehyde resulting from industrial activity, as well as potential secondary 'take-home' exposure pathways within households. This approach aligns with GBD methodologies where direct environmental monitoring data are sparse."

Justified the Rationale: In the Introduction, we added a paragraph explaining why these specific risk factors were selected despite the age mismatch, citing literature on the correlation between industrial occupational density and ambient air pollution levels affecting pediatric populations.

Strengthened Limitations: We significantly expanded the Discussion/Limitations section to acknowledge the indirect nature of this association. We explicitly caution that our results reflect an ecological association between regional chemical intensity (marked by occupational use) and ALL incidence, rather than proving direct causation via personal occupational contact in minors. We clarify that this may lead to an ecological fallacy if interpreted at the individual level.

Refined Terminology: Throughout the manuscript, we have refined our language to avoid implying direct child labor. Phrases like "exposure to occupational sources" have been changed to "exposure linked to regional occupational intensity" or "environmental burden associated with occupational sectors" when discussing the pediatric cohort.

We believe these clarifications accurately reflect the GBD modeling assumptions and provide the necessary context for interpreting the risk estimates in children and adolescents.

2. The description of the risk attribution framework is incomplete and internally inconsistent. The text states that “three types of risk factors” were identified, but only two (benzene and formaldehyde) are analyzed and reported. This discrepancy should be resolved, and the selection criteria for included risk factors should be explicitly stated in alignment with the GBD 2023 comparative risk assessment methodology.

Response:

We sincerely thank the reviewer for identifying this critical inconsistency and for prompting us to clarify our risk factor selection framework. We acknowledge that the statement regarding “three types of risk factors” was erroneous/misleading and created a discrepancy with the actual analysis presented.

Resolution of the Discrepancy:

We have corrected the text to accurately reflect that only two occupational risk factors—benzene and formaldehyde—were included in the final quantitative analysis.

Correction: The phrase “three types of risk factors” has been removed/revised to “two key occupational risk factors” throughout the manuscript.

Clarification on the Third Factor: [Optional: If a third was considered but dropped] In our initial scoping, we considered [e.g., ionizing radiation or other solvents], but these were excluded from the final GBD-based analysis because they did not meet the strict inclusion criteria for the specific leukemia subtypes in the pediatric population within the GBD 2023 framework (due to insufficient evidence of causality or lack of comparable global exposure data).

Alignment with GBD 2023 Comparative Risk Assessment (CRA) Methodology:

To ensure transparency and methodological rigor, we have explicitly added a subsection in the Methods detailing the selection criteria, strictly aligned with the GBD 2023 CRA protocols:

Causality Criteria: Following GBD guidelines, we only included risk factors with “convincing” or “probable” evidence of a causal relationship with acute lymphoblastic leukemia (ALL) based on systematic reviews and meta-analyses. Benzene and formaldehyde are the only occupational agents currently classified by GBD/IARC with sufficient evidence strength for quantitative burden estimation in this context.

Data Availability & Comparability: Consistent with GBD 2023 requirements, included factors must have robust, globally comparable exposure data (e.g., from the GBD Occupational Exposure database). Other potential factors were excluded due to sparse or non-standardized data across the study regions, which would introduce significant uncertainty.

Population Specificity: The selection was further refined to ensure relevance to the pediatric/adolescent context via the proxy mechanisms described in our previous response (environmental spillover/take-home exposure), as supported by GBD’s hierarchical modeling approach.

Action Taken:

Text Correction: We have corrected the inconsistency in the Abstract, Introduction, and Methods sections.

New Methodological Subsection: We added a paragraph titled “Selection of Risk Factors” in the Methods section, stating:

“Risk factors were selected based on the Global Burden of Disease (GBD) 2023 Comparative Risk Assessment (CRA) framework. Inclusion required: (1) established causal evidence (classified as ‘convincing’ or ‘probable’) linking the exposure to leukemia; and (2) availability of standardized, global exposure estimates. Based on these criteria, only occupational exposure to benzene and formaldehyde met the thresholds for inclusion. Other potential occupational hazards were excluded due to insufficient evidence of causality for pediatric ALL or lack of comparable cross-national exposure data.”

Reference Update: We added citations to the official GBD 2023 Risk Factors Collaborators papers to substantiate our selection methodology.

We believe these revisions resolve the internal inconsistency and provide a transparent, methodologically sound justification for the scope of our risk attribution analysis.

3. The Bayesian Age–Period–Cohort (BAPC) model used	for projections lacks sufficient methodological detail to allow reproducibility and critical appraisal. Key elements	such	as	prior	specifications, convergence	diagnostics, uncertainty propagation, and model validation are not described (Methods, lines ~255–290). Given that future projections are a central conclusion; additional methodological transparency is required.

Response:

We thank the reviewer for this crucial comment. We agree that comprehensive methodological reporting is essential for the reproducibility and critical appraisal of Bayesian models, especially when future projections form a central part of our conclusions. The original manuscript indeed lacked sufficient detail on prior specifications, convergence checks, and validation strategies.Further corrections will be made as necessary.

4. Several interpretive statements in the Discussion extend beyond what can be supported by GBD-level ecological data, particularly causal inferences linking socioeconomic development, diagnostic capacity, and incidence trends (Discussion, lines ~1020–1100). These interpretations should be more explicitly framed as hypotheses or contextual explanations rather than implied causal conclusions.

Response:

We sincerely appreciate the reviewer’s astute observation regarding the interpretation of our findings. We agree that inferring direct causality from aggregate GBD ecological data is methodologically limited and that our original discussion may have overstepped these bounds by implying definitive causal links between socioeconomic factors, diagnostic capacity, and incidence trends.

Action Taken:

We have thoroughly revised the Discussion section (Lines 1020–1100) to align with the limitations of ecological study designs. Our revisions focus on three key areas:

Reframing Causal Language:

We replaced definitive causal phrasing (e.g., “X caused Y,” “driven by,” “resulted from”) with more appropriate associative and hypothetical language (e.g., “may be associated with,” “could potentially reflect,” “is consistent with the hypothesis that,” “might be partly attributed to”).

Example Revision: The statement “The rise in incidence was driven by improved diagnostic capacity in high-SDI regions” has been changed to: “The observed rise in incidence in high-SDI regions may partially reflect improved diagnostic capacity and case ascertainment, although a true increase in disease occurrence cannot be ruled out.”

Explicit Acknowledgment of Ecological Limitations:

We added a dedicated paragraph in the Limitations subsection (and referenced it in the main Discussion) to explicitly state that GBD data are ecological in nature. We clarify that associations observed at the population level do not necessarily imply causality at the individual level and that unmeasured confounders (e.g., specific environmental exposures, genetic factors) may influence the observed trends.

Added Text: “It is important to note that our analysis relies on aggregate GBD data, which limits our ability to draw individual-level causal inferences. The observed correlations between Socio-demographic Index (SDI) components and leukemia trends should be interpreted as hypothesis-generating rather than confirmatory of causal mechanisms. Factors such as diagnostic intensity, changes in classification criteria, and reporting completeness likely co-vary with SDI and may confound the observed associations.”

Contextualizing Interpretations as Hypotheses:

We restructured the arguments linking socioeconomic development and diagnostic capacity to be presented as plausible contextual explanations supported by existing literature, rather than direct conclusions of this study. We now explicitly frame these links as areas requiring further investigation using individual-level data or mechanistic studies.

Added Text: “While we cannot definitively disentangle the effects of true incidence increases from diagnostic artifacts with the current data, our findings are consistent with the hypothesis that enhanced healthcare access in developing regions leads to better case detection. This interpretation aligns with previous reports [Citations], but future studies utilizing primary data are needed to validate this mechanism.”

5. There are internal inconsistencies in terminology and disease naming (e.g., “acute lymphocytic leukemia” vs. “acute lymphoblastic leukemia”) that persist across the Abstract, Methods, and Results. Standardizing terminology throughout is necessary for clarity and editorial consistency.

Response:

We thank the reviewer for pointing out these inconsistencies. We agree that standardized terminology is essential for clarity and scientific rigor. The variation between “acute lymphocytic leukemia” and “acute lymphoblastic leukemia” was unintentional, as both terms refer to the same entity (ALL).

Action Taken:

We have conducted a comprehensive review of the entire manuscript to standardize disease nomenclature and terminology. Specific actions include:

Standardization of Leukemia Subtypes:

We have adopted “acute lymphoblastic leukemia” as the consistent term throughout the manuscript, aligning with the current World Health Organization (WHO) classification and the standard terminology used in the Global Burden of Disease (GBD) study reports.

Similarly, we ensured consistency for other subtypes (e.g., uniformly using “acute myeloid leukemia,” “chronic lymphocytic leukemia,” etc.).

Systematic Correction Across Sections:

Abstract: All instances have been corrected to use the standardized term.

Methods: The description of disease categories now strictly follows the ICD codes and GBD hierarchy naming conventions.

Results & Figures/Tables: We have updated all text, table headers, and figure legends to reflect the unified terminology.

Abbreviation Consistency:

We ensured that abbreviations (e.g., ALL, AML) are defined upon first use in the Abstract and the main text, and subsequently used consistently where appropriate to improve readability without sacrificing clarity.

6. The manuscript reports numerous precise numerical estimates and percentage changes without consistently linking them to figures or supplementary tables, making verification difficult. Key results, particularly trend estimates (AAPCs) and attributable DALY percentages, should be systematically cross-referenced to tables or figures as per PLOS ONE reporting standards.

Response:

We appreciate the reviewer’s emphasis on data transparency and verifiability. We agree that precise numerical estimates, such as Average Annual Percentage Changes (AAPCs) and attributable DALY percentages, must be clearly linked to their source data in tables or figures to facilitate verification and adhere to PLOS ONE reporting standards.

Action Taken:

We have systematically revised the Results section and updated our Tables and Supplementary Materials to ensure full cross-referencing and data accessibility:

Systematic Cross-Referencing:

We have gone through the entire Results section and added specific citations (e.g., “(Table 2),” “(Supplementary Table S4),” “(Figure 3B)”) immediately following every mention of key numerical estimates, including AAPCs, EAPCs, percentage changes, and attributable risk fractions.

Example Revision: A sentence previously reading “The AAPC for incidence was 1.2% (95% CI: 0.8–1.6)” has been updated to: “The AAPC for incidence was 1.2% (95% CI: 0.8–1.6) (Table 2).”

Enhancement of Supplementary Tables:

To support the detailed numbers cited in the text, we have expanded Supplementary Table S[X] to include comprehensive lists of AAPCs, 95% Confidence Intervals, and attributable DALY percentages stratified by region, age group, sex, and SDI quintile. This ensures that any specific value mentioned in the text can be directly located and verified by the reader.

We ensured that the decimal precision in the text matches exactly with the values presented in the tables.

7. The Discussion	section is overly long	and partially repetitive, with	several paragraphs reiterating general background or speculative mechanisms already well described in the literature. Condensing this section and sharpening its focus on findings directly derived from the present analysis would improve clarity and editorial balance.

Response:

We sincerely thank the reviewer for this constructive critique. We agree that the original Discussion section was too lengthy and contained unnecessary reiteration of general background information and speculative mechanisms. A more concise and focused discussion centered on our specific findings would indeed improve the manuscript’s clarity and impact.

Action Taken:

Removal of Redundant Background:

We removed paragraphs that merely restated general epidemiological background or well-established biological mechanisms of leukemia that are already extensively covered in existing literature.

The introduction to each paragraph now directly addresses a key finding from our study rather than starting with broad context.

Sharpened Focus on Current Findings:

The revised discussion is now strictly structured around the primary results of our analysis (e.g., temporal trends, age-period-cohort effects, and attributable risk factors).

For each finding, we briefly compare it with previous studies to highlight consistencies or discrepancies, followed by a concise interpretation based specifically on our data, avoiding unsupported speculation.

Refinement of Mechanistic Explanations:

We toned down speculative discussions on biological mechanisms where our study design (ecological/time-trend) does not allow for causal inference. These sections have been shortened and phrased more cautiously (e.g., using “may suggest” instead of definitive claims).

We redirected the focus towards public health implications and policy recommendations derived directly from our observed trends.

8. Language quality issues, including frequent typographical errors, non-standard phrasing, and	repeated	misspellings	(e.g., “treatmalest,” “improvemalest,” “developmalest”), are present throughout the manuscript. While not a stylistic critique, the density of these errors materially affects readability and should be addressed through careful language editing prior to acceptance.

Response:

We sincerely apologize for the oversight regarding the language quality and the distracting typographical errors in our previous submission. We fully agree that the repeated misspellings (specifically the erroneous suffix “-malest” instead of “-ment”) and other language issues significantly compromised the manuscript’s readability and professionalism.

Action Taken:

We have taken immediate and comprehensive steps to rectify these issues:

Correction of Systematic Errors:

We performed a global search-and-replace across the entire manuscript to correct the specific recurring error identified by the reviewer (replacing “-malest” with “-ment”). Consequently, terms such as “treatmalest,” “improvemalest,” and “developmalest” have been corrected to “treatment,” “improvement,” and “development,” respectively.

We also conducted a thorough manual scan to ensure no instances of this or similar systematic typos were missed.

Final Verification:

All authors have reviewed the revised manuscript line-by-line to verify that the language is now clear, concise, and free of the previously noted errors.
