# Supplementary material for: Burden of acute lymphoblastic leukemia in children and adolescents in low- and middle-income countries from 1990 to 2023 and projections to 2050: A systematic analysis from the global burden of disease study 2023
Source: PLoS One. 2026 Jun 2;21(6):e0350223. doi: 10.1371/journal.pone.0350223 (PMC13229300; doi:10.1371/journal.pone.0350223)
Supplement: Attachment — Submitted filename: Response to Reviewers 4.docx [file pone.0350223.s011.docx]

1. Abstract and introduction are well corrected.

Response:

We sincerely thank the reviewer for the positive feedback on the abstract and introduction. We are glad that our revisions have met the expectations and improved the clarity and quality of these sections.

2. For Methods section, you can mention in the manuscript why you focused specifically on occupational benzene and formaldehyde (which was written in Response to Reviewers).

Response:

We thank the reviewer for the suggestion to clarify in the manuscript why we focused specifically on occupational benzene and formaldehyde exposure. In response, we have added the following sentence to the “Attributable burden analysis” subsection of the Methods section:

“These two risk factors were selected because they are the only occupational chemical exposures with sufficient epidemiological evidence to be included in the GBD comparative risk assessment framework for ALL, and they represent modifiable exposures of potential public health relevance in LMIC settings.”

This addition explicitly states the rationale for selecting these two risk factors, addressing the reviewer’s concern and improving the transparency of our methodological choices. We believe this revision further strengthens the clarity and reproducibility of the Methods section.

3. Please carefully review the manuscript again to make sure there are no grammatical errors or typographical mistakes throughout the text. For example:
a. Lines 145–146: “For males, it was 3.53 (95% UI: 2.32–5.47), and for females, it was 2.49 (95% UI: 1.62–3.86) per .” — this sentence is incomplete.
b. Lines 304–308: “In contrast, the decline in age-standardised incidence rate (ASIR), age-standardised mortality rate (ASMR), and age-standardized disability-adjusted life year (DALY) rate (ASDR) of acute lymphocytic leukemia in low-income countries during the same period was relatively small, which may reflect that these countries' health status.” — this sentence is also incomplete.

Response:

We thank the reviewer for the careful proofreading and for pointing out specific typographical errors. We have thoroughly reviewed the entire manuscript and corrected all identified grammatical and typographical mistakes.

4. Please ensure that abbreviations are used consistently throughout the manuscript. Some abbreviations are repeated excessively, which may confuse readers. In addition, there is inconsistency in the use of “acute lymphoblastic leukemia” versus “acute lymphocytic leukemia.” Please standardize the terminology.

Response:

We thank the reviewer for this careful observation. We have thoroughly reviewed the entire manuscript to ensure consistent use of abbreviations and terminology.

5. The Discussion section is overly long and contains redundant content (particularly in the first paragraph), which obscures the main message. I recommend revising this section to be more concise and focused.

Response:

Response to the reviewer:

We thank the reviewer for this valuable suggestion. We agree that the original Discussion section was overly long, with redundant content in the first paragraph that obscured the main message. We have substantially revised the Discussion as follows:

1. Condensed the first paragraph:

The original first paragraph provided a broad restatement of background information that had already been covered in the Introduction. We have removed this redundancy and now start the Discussion directly with the interpretation of our key findings. The revised opening paragraph now reads:

*“Our study revealed a clear socioeconomic gradient in ALL burden in LMICs from 1990 to 2023. In 2023, age-standardized incidence, mortality, and DALY rates all increased progressively from low-income to upper-middle-income countries. This pattern likely reflects a combination of genuine risk differences and diagnostic capacity.”*

2. Focused on interpretation of findings:

We have refocused the entire Discussion to emphasize interpretation of our key findings rather than providing a broad overview of global trends. Redundant descriptions of results that are already presented in the Results section have been removed.

3. Improved conciseness:

We have shortened the Discussion by approximately 30% by:

Removing redundant background statements

Eliminating repetitive descriptions of results

Consolidating overlapping content

Streamlining the risk factor attribution discussion

4. Reorganized structure:

The Discussion is now organized into four clear subsections for better readability:

Interpretation of key findings

Temporal trends and future projections

Risk factor attribution

Study limitations

We believe these revisions have made the Discussion more concise, focused, and easier to follow while preserving all essential interpretations. We are grateful for the reviewer’s guidance in improving this section.

6. In the Discussion, the authors note that in high- and middle-income countries, the highest incidence and mortality rates are concentrated in the <1-year age group, whereas in LICs and LMICs, these rates are highest in the 2–4-year age group. However, the manuscript does not provide a discussion of the possible reasons for this difference. Please elaborate on this point.

7. Similarly, the authors state that the incidence rate is highest among children aged <5 years in low- and middle-income countries and regions, but this observation is not further discussed. Please provide potential explanations or relevant context.

Response:

We thank the reviewer for these important observations. We agree that the age-specific patterns of ALL burden—particularly the shift in peak age from infancy in UMICs to 2–4 years in LICs and LMICs, as well as the high incidence in children under 5 years—require further discussion.

In response, we have added the following concise explanation to the “Interpretation of key findings” subsection of the Discussion:

*“This age shift may be explained by the delayed infection hypothesis (Greaves' hypothesis): reduced early-life microbial exposure in UMICs delays ALL onset to infancy, whereas early infections in LICs/LMICs trigger earlier transformation at 2–4 years . Diagnostic capacity also contributes—UMICs more readily detect aggressive infant ALL , while diagnostic delays in LICs/LMICs shift the apparent peak to older ages . Additionally, the highest incidence in children under 5 years reflects the global biological peak of ALL in the first five years of life, driven by immune development and hematopoietic proliferation windows.”*

We believe this addition provides a clear and concise explanation for the observed age-specific patterns, addressing both the cross-country differences in peak age and the consistent under-5 peak. The revised Discussion now directly links these patterns to biological mechanisms (Greaves' hypothesis), health system factors (diagnostic capacity), and established epidemiological knowledge (global peak in under-5 children).

We are grateful for the reviewer’s suggestion to elaborate on these important points, which has strengthened the interpretative depth of our Discussion.
