# Supplementary material for: Burden of acute lymphoblastic leukemia in children and adolescents in low- and middle-income countries from 1990 to 2023 and projections to 2050: A systematic analysis from the global burden of disease study 2023
Source: PLoS One. 2026 Jun 2;21(6):e0350223. doi: 10.1371/journal.pone.0350223 (PMC13229300; doi:10.1371/journal.pone.0350223)
Supplement: Attachment — Submitted filename: Reviewer Comments.docx [file pone.0350223.s006.docx]

**The Burden of Acute Lymphoblastic Leukemia and Associated Risk Factors in Children**

**and Adolescents of Low- and Middle-Income Countries from 1990 to 2023**

**Title**

The title is overly broad and implies a depth of causal inference regarding “associated risk factors” that is not supported by the analyses performed.

The study is fundamentally descriptive and limited to GBD-attributable risk modeling, yet the title suggests a comprehensive assessment of risk factors for ALL in children and adolescents. This overstatement risks misleading readers regarding the scope and strength of the findings.

**Abstract**

The abstract contains several conceptual and technical issues.

The description of methods is imprecise, with unclear terminology (e.g., “whole life years” instead of DALYs) and grammatical inaccuracies that obscure meaning.

The emphasis on occupational benzene and formaldehyde exposure in children and adolescents is not adequately contextualized, raising immediate concerns about biological plausibility and exposure pathways.

The results section of the abstract selectively highlights findings without conveying the very small attributable fractions reported, potentially exaggerating the public health relevance of these risk factors.

**Introduction**

The introduction is lengthy but lacks focus and critical synthesis.

While background information on ALL is extensive, the rationale for focusing on occupational exposures in a pediatric population is weak and insufficiently justified.

Key assumptions, such as indirect exposure of children to occupational carcinogens, are implied rather than explicitly stated or supported by evidence.

The introduction also fails to clearly articulate how this study meaningfully advances beyond previously published GBD-based analyses of childhood leukemia.

**Methods**

The selection of risk factors is not adequately justified, particularly given that only occupational benzene and formaldehyde exposure are ultimately analyzed despite the GBD framework including many other environmental and behavioral risks.

The application of occupational exposure models to children and adolescents is not explained, nor is it clarified whether these estimates represent direct exposure, parental exposure, or modeled population-level associations.

The Bayesian Age-Period-Cohort projections assume continuity of past trends without sufficient discussion of uncertainty or structural limitations, especially in LMIC settings where surveillance quality has changed substantially over time.

**Results**

The results section is excessively descriptive and difficult to follow, with frequent repetition of numerical estimates that add little interpretive value.

Several claims, particularly those highlighting benzene as the “primary” risk factor, are not contextualized by the extremely small attributable proportions reported, which limits their epidemiological significance.

Geographic comparisons are presented without sufficient discussion of data quality heterogeneity across countries, and some country-level rankings may reflect registry completeness rather than true disease burden.

Figures and tables are numerous but not always well-integrated into the narrative, reducing their interpretive usefulness.

**Discussion**

The discussion overinterprets several findings and at times moves beyond what can reasonably be inferred from GBD modeling.

Statements implying causality or policy prioritization of occupational exposures for childhood ALL are not sufficiently supported, given the marginal attributable burden and indirect exposure pathways.

The discussion also introduces concepts such as liquid biopsy and early molecular detection that are not directly linked to the study’s data or analyses, creating conceptual drift.

While limitations are acknowledged, they are not fully explored, particularly regarding exposure misclassification, ecological inference, and the appropriateness of occupational risk attribution in pediatric populations.

**Conclusions**

The conclusions are broader and more prescriptive than warranted by the results. Recommendations related to occupational exposure control, while important in general, are not convincingly supported as priority interventions for reducing childhood ALL burden based on the data presented.

The conclusion would benefit from greater restraint and clearer alignment with the descriptive nature of the study.

**References**

The reference list contains several weaknesses, including redundancy, inconsistent formatting, and reliance on older or tangentially relevant citations.

Some references are repeated, while others do not directly support the specific claims made in the text. The balance between recent high-quality evidence and historical references is suboptimal, particularly in sections discussing risk factors and mechanistic interpretations.
